# Protein-Based Silver Nanoparticles: Synthesis, Characterization, Administration, and Nanomedicine Applications

**DOI:** 10.1155/ijbm/5533798

**Published:** 2025-05-26

**Authors:** Syed Muhammad Hasan, Saadat Hussain, Muhammad Yousuf, José Agustín Tapia-Hernández, Daim Asif Raja

**Affiliations:** ^1^Memon Medical Institute Hospital, Karachi, Pakistan; ^2^H.E.J Research Institute of Chemistry, ICCBS, University of Karachi, Karachi 75270, Pakistan; ^3^Department of Biomedical and Biological Sciences, Sohail University, Karachi 74000, Pakistan; ^4^Department of Food Research and Graduate Program, University of Sonora, C.P. 83000, Hermosillo, Sonora, Mexico

**Keywords:** chronic diseases, nanomedicine, protein-based nanoparticles, routes of administration, silver

## Abstract

Nanotechnology has emerged as a transformative field in recent years, greatly impacting medicine and healthcare with innovative solutions for complex diseases. Among these advancements, protein-based metal nanoparticles have shown exceptional promise in treating chronic illnesses, owing to their high biocompatibility, biodegradability, customizable surface properties, and precise drug delivery capabilities. Recent studies have highlighted advancements in targeting efficiency and controlled release, alongside the ability of protein-based metal nanoparticles to bypass the first-pass metabolism, enhancing bioavailability through novel administration routes. Cutting-edge research has also focused on functionalizing protein nanostructures with therapeutic metal ions, particularly silver, with a longstanding antimicrobial and anti-inflammatory history. New combinations of silver with protein-based nanoparticles are now showing significant potential in managing chronic and life-threatening conditions. This review provides a comprehensive overview of the latest synthesis methods, toxicity assessments, therapeutic applications, administration pathways, and advanced characterization techniques for protein-based silver nanoparticles, addressing the evolving landscape of nanomedicine.

## 1. Introduction

In recent decades, nanotechnology has revolutionized multiple scientific and medical fields, with nanomedicine being one of the most prominent areas of advancement. A significant breakthrough in this domain is the development of protein-based silver nanoparticles (Ag NPs) [1]. These nanoparticles, known for their unique properties, combine the biocompatibility of proteins with silver's potent antimicrobial and anticancer capabilities, opening new pathways for innovative and effective medical treatments. The synthesis of protein-based Ag NPs is a sophisticated process that integrates chemical and biological techniques to produce structures with specific and controlled characteristics. This approach not only enhances the stability and functionality of the nanoparticles but also allows for greater precision in their size and shape. Proteins act as reducing and stabilizing agents during synthesis, ensuring that the resulting nanoparticles possess optimal properties for medical applications [2].

Characterization of nanoparticles is an essential and crucial step in gaining a deep understanding of their physical and chemical properties. This process not only confirms the successful synthesis of nanoparticles but also provides a detailed insight into their morphology, size, composition, and structure. To achieve this, a range of advanced techniques including transmission electron microscopy (TEM), infrared (IR) spectroscopy, and energy-dispersive spectroscopy (EDS) are employed. TEM is particularly valuable for observing the shape and size of nanoparticles at a nanometric scale, producing high-resolution images that reveal intricate structural details. IR spectroscopy helps identify the various chemical functionalities present on the surface of nanoparticles, essential for understanding their reactivity and stability. EDS complements these analyses by offering precise information on the elemental composition of nanoparticles, identifying the elements present and their distribution [3]. These studies are not only critical for verifying the proper fabrication of nanoparticles but also play a significant role in predicting and evaluating their biological behavior. By understanding how these nanoparticles interact with living systems, scientists can anticipate their toxicity, biodistribution, and efficiency in biomedical applications such as drug delivery or gene therapy [4].

Metal-based nanoparticles such as gold, silver, and iron oxide NPs are commonly synthesized through chemical reduction, where metal salts are reduced to produce nanoscale particles [5]. Methods like green synthesis, using plant extracts or microbial agents, have been developed to reduce the toxicity and environmental impact of metal NPs. Metal-based NPs are primarily utilized for imaging and antimicrobial applications, as well as in targeted cancer therapies due to their ease of functionalization, high stability, and ability to facilitate localized treatments. However, potential cytotoxicity and long-term accumulation in tissues remain concerns, particularly in sensitive biomedical applications [6]. Polymer-based nanoparticles are synthesized through emulsion, nanoprecipitation, and solvent evaporation techniques. They offer great versatility, as polymers can encapsulate both hydrophilic and hydrophobic drugs, control drug release rates, and reduce toxicity. Polymeric NPs are widely used in controlled drug delivery and gene therapy, enabling precise delivery to target sites and minimizing systemic side effects. Despite these benefits, some polymers may degrade into toxic byproducts, posing challenges in long-term use [7]. Lipid-based nanoparticles like liposomes and solid lipid NPs (SLNs) are typically created using high-pressure homogenization, thin-film hydration, and micro-emulsion methods. These are biocompatible, allows the encapsulation of hydrophobic drugs, and can merge with cell membranes for effective intracellular delivery. Lipid NPs are heavily utilized in cancer therapies and vaccine delivery platforms (e.g., mRNA vaccines), as they can be modified for controlled release. However, they can sometimes lack stability and are sensitive to storage conditions, which can limit their shelf life and practical application [8].

In contrast, protein-based nanoparticles represent a promising alternative due to their high biocompatibility, biodegradability, and low toxicity. Commonly synthesized through desolvation, emulsification, self-assembly, or cross-linking techniques, protein NPs provide natural binding sites for drugs, allowing high drug-loading capacities and sustained release profiles. Proteins like albumin are particularly advantageous as carriers because they can encapsulate both hydrophilic and hydrophobic drugs while maintaining structural stability. Additionally, protein NPs bypass the first-pass metabolism and offer targeted drug delivery capabilities, enhancing bioavailability and reducing systemic side effects [9].

Unlike metal-based NPs, which can raise cytotoxicity concerns, protein-based NPs are naturally biocompatible and degrade safely in biological systems [10]. Compared to polymeric NPs, proteins offer more effective binding sites, enhancing drug encapsulation and stability. Additionally, lipid-based NPs are more prone to stability issues, while protein NPs maintain structural integrity in a variety of environments [11].

Despite their benefits, silver-based nanomaterials still present risks of toxicity. The release of silver ions from Ag NPs creates oxidative stress which produces damaging effects on both microbial and mammalian cells that eventually result in cellular death. Scientific evidence suggests Ag NPs lead to cytotoxic effects by harming mitochondrial function while producing reactive oxygen species that block enzymatic pathways. Long-term exposure to silver-based nanomaterials produces genotoxic effects and inflammatory responses which require a thorough analysis of safety parameters for their dosage and usage [12].

The fundamental requirement of silver-based nanomaterials is their ability to interact well with biological systems. The long-standing medical use of silver in wound treatments and dental applications remains a concern because silver can trigger immune system reactions and impair natural cellular operations. The interaction between Ag NPs and proteins and cell membranes disrupts cellular functions, so researchers need to develop surface modification or encapsulation techniques to improve their biological compatibility. Scientists work to embed Ag NPs with protective biocompatible polymers or biomolecules or peptides to minimize negative side effects and enhance therapeutic results [13].

Bioaccumulation stands as a significant environmental problem that silver-based nanomaterials create. After their release into biological or environmental systems, Ag NPs demonstrate persistence which enables them to build up in tissues, thus generating lasting ecological and health challenges. Multiple studies confirm Ag NPs tend to collect in liver organs together with kidneys and spleen which leads to possible systemic toxic effects. Aquatic ecosystems are negatively affected by Ag NPs because they alter microbial diversity, create disruptions in food chain relationships, and lead to silver accumulation in aquatic organisms. Extensive risk assessment together with proper dosing approaches and biodegradable or controlled-release nanomaterial development needs to be developed to solve these problems [14].

The applications of protein-based Ag NPs in nanomedicine are extensive and promising. These nanoparticles have been widely studied in the treatment of bacterial infections and cancerous diseases, leveraging their antimicrobial and cytotoxic properties [15, 16]. For instance, Ag NPs can penetrate the cellular membranes of bacteria and cancer cells, disrupting their vital functions and inducing cell death. Moreover, their ability to be functionalized with specific therapeutic molecules enables them to selectively target cells of interest and deliver drugs in a controlled manner. This targeted delivery capability enhances treatment efficacy by concentrating therapeutic effects while reducing side effects in healthy tissues [17]. To sum up, protein-based Ag NPs represent an innovative convergence of biology and nanotechnology with immense potential to transform modern medicine. Therefore, the objective of this review article is to provide a comprehensive analysis of protein-based Ag NPs, focusing on their synthesis methods, characterization techniques, mechanisms of action, and diverse applications in nanomedicine.

## 2. Approaches for Making Nanoparticles

The preparation of nanoparticles involves either a “top-down” or a “bottom-up” approach. In the “top-down approach,” nanoparticles are synthesized by breaking down large molecules into smaller ones by applying various physical and chemical methods. In “bottom-up synthesis,” the smaller entities are combined to form nanostructures which eventually lead to the formation of the desired product. The bottom-up synthesis is highly dependent on chemical and biological processes for production [3].

Nanoparticles are defined as particles having a size between 1 and 100 nm. They are considered simple molecules; however, they are complex in nature and have extensive chemical integration. Nanoparticles can absorb light, dissolve, and possess a high surface area to volume ratio that contributes to their unique behavior. Surface chemistry is an integral part of nanoparticles which is completely different than the nanoparticle core. A nanoparticle that is exposed to external factors should have correspondent functional groups adhered to its exterior surface, which may include short peptides, glycoproteins, and metal ions [18–20]. Moreover, many molecules modify the surface chemistry and functionalization of nanoparticles that induce those relevant characteristics. To provide stability, nanoparticles are coated to prevent aggregation in solutions and their immediate loss of structure. These surface coatings act as a surfactant which forms Van der Waals interactions with the surface molecules. Summarizing key points, a nanoparticle can be divided morphologically into three subdivisions: (i) Surface: It may be functionalized with specific functional groups, metal ions, surfactants, or polymers attached/adhered/adsorbed onto its surface. (ii) Shell material: It can be added intentionally and is utilized for the encapsulation of nanomaterials. (iii) Core: A major part of nanoparticle because of its major therapeutic applications [21].

Nanoparticles exhibit incredible interrelations of electric currents and magnetism, surface chemistry, and catalytic properties because of their remarkably low volume-to-surface area ratio. Metallic nanoparticles like silver exhibit high chromaticity due to their surface plasmon resonance (SPR) phenomenon. The electrons present in the metal ions are in continuous resonance that displays chromaticity in the IR and UV-visible (UV-vis) regions [22].

## 3. Ag NPs and Their Synthesis

Silver (Ag), a lustrous transition metal in Group 11 of the periodic table, possesses an atomic number of 47 and an atomic weight of 107.868 g/mol. Its characteristic smooth surface and high electrical and thermal conductivity have made it a valuable material throughout history. It has been widely used in various forms, including solvents, foils, currency, utensils, surgical sutures, pharmaceuticals, and colloids. Notably, silver exhibits potent antimicrobial, antifungal, and anti-inflammatory properties, making it a promising therapeutic agent for a range of medical conditions [23].

Although Ag NPs can theoretically exist in pure form in solution, they are more commonly found as composites, nanoparticles, and agglomerated nanoparticles. These particles can vary significantly in size, ranging from smaller than 2 nm to larger than 500 nm. Larger particles, typically within the range of 2–500 nm, are referred to as colloidal silver and can be observed microscopically dispersed throughout the solution [24].

The contemporary research landscape is characterized by a strong focus on the applications of nanoparticles across various scientific fields. Ag NPs have emerged as an important class of nanomaterials due to their cost-effectiveness, ease of synthesis, and high efficiency. The unique properties of these nanomaterials have driven extensive research and development in electronics, nanomedicine, biomaterials, energy, and food science [25].

**Figure 1:**
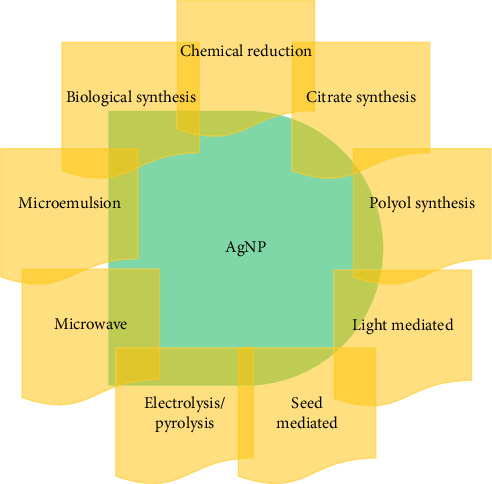
Representation of different synthetic procedures involved in the preparation of silver nanoparticles.

The preparation of Ag NPs involves several methods in for controlling morphology (Figure 1). These methods are cost-effective and environment-friendly, provide better results with less wear and tear, and display a great abundance of plasmon excitations. Furthermore, the scientific community is now focused on the future applications of Ag NPs because of their capability to absorb light in the visible spectrum and display amazing relevant characteristics [26].

### 3.1. Chemical Reduction Synthesis

The most used method to synthesize Ag NPs is chemical reduction synthesis in which different reducing agents are used either organic or inorganic to reduce silver ions from (Ag^+^) to Ag (0) [27]. The most frequently used reducing agents involve sodium borohydride, tri-sodium citrate, and ascorbic acid. These reducing agents reduce the Ag^+^ to Ag^0^ [28]. For the preparation of Ag NPs, the ideal source of silver and the favored precursor is silver nitrate (AgNO_3_) because it is convenient to use, possesses no potential hazards, and is easily available. However, studies have reported the inadequate use of several reducing agents during the synthesis of Ag NPs may lead to the aggregation of nanoparticles in aqueous form [29]. To prevent the aggregation of nanosized silver particles, stabilizing agents are being used such as liposomes, flavonoids, polysaccharides, synthetic polymers, and proteins. Liposomes can encapsulate Ag NPs within their lipid bilayers, providing a protective environment that prevents agglomeration. Their biocompatibility and ability to facilitate controlled release make them ideal for drug delivery applications [30]. Flavonoids, which are natural antioxidants, can stabilize Ag NPs through their functional groups, such as hydroxyl and carbonyl groups. These compounds not only reduce silver ions to form nanoparticles but also provide a stabilizing layer that prevents aggregation [31]. Polysaccharides like chitosan and starch stabilize Ag NPs by forming a physical barrier around the nanoparticles [32, 33]. Their high molecular weight and functional groups contribute to effective stabilization and enhance biocompatibility. Synthetic polymers such as polyethylene imine (PEI) and polyvinylpyrrolidone (PVP) are commonly used to stabilize Ag NPs as they provide steric stabilization, preventing aggregation through repulsive forces created by the polymer chains [34]. Similarly, proteins can stabilize Ag NPs by adsorbing them onto their surfaces, providing steric and electrostatic stabilization. These interactions can also enhance the biocompatibility of Ag NPs for biomedical applications [35]. For instance, Sergeev et al. synthesized protein-based Ag NPs in deionized water by using silver nitrate (AgNO_3_) as a metal ion source and sodium borohydride (NaBH_4_) as a reducing agent. The solution turned bright yellow-brownish in color after the complete reduction of silver ions on continuous stirring at room temperature in acidic pH [36]. Chekin and Ghasemi prepared Ag NPs using ascorbic acid as the reducing agent by dissolving gelatin in deionized water on continuous stirring followed by the addition of AgNO_3_ and ascorbic acid. The solution is kept in a water bath, and the resultant product is obtained at 500°C after 8 h [37].

### 3.2. Citrate Synthesis

The citrate reduction method is a widely used approach for synthesizing Ag NPs due to its simplicity and cost-effectiveness. This method involves the reduction of silver nitrate (AgNO_3_) by sodium citrate, which also acts as a stabilizing agent to prevent nanoparticle aggregation. The reaction typically occurs by adding sodium citrate dropwise into a boiling silver nitrate solution, leading to a color change from pale yellow to grayish-brown, indicating the formation of Ag NPs [37]. This method provides advantages such as ease of execution, reproducibility, and the ability to produce uniform nanoparticles. However, drawbacks include potential particle aggregation if not properly stabilized and the necessity of precise control over reaction parameters such as temperature and reactant concentrations to achieve the desired particle size [37]. Recent studies have optimized the citrate reduction method to enhance nanoparticle stability and functionalization. For example, Zhou and Wang synthesized Ag NPs by adding sodium citrate into a boiling AgNO_3_ solution and observed that the cooling process significantly influenced particle uniformity. The researchers confirmed successful nanoparticle formation through UV-vis spectroscopy, which displayed a characteristic SPR peak at 425 nm [38]. These recent developments demonstrate the continuous refinement of the citrate reduction method to improve Ag NP synthesis for biomedical and nanotechnology applications.

### 3.3. Polyol Synthesis

The polyol method is a widely utilized technique for synthesizing Ag NPs due to its simplicity and effectiveness in producing uniform particles. This method involves the reduction of silver salts in a polyol, such as ethylene glycol (EG) or glycerol, under controlled conditions. In this process, the polyol not only acts as a reducing agent but also helps stabilize the resulting nanoparticles, preventing agglomeration. By adjusting parameters like temperature, silver precursor concentration, and reaction time, researchers can control the size and morphology of the Ag NPs. Recent studies have shown that functionalizing Ag NPs synthesized via the polyol method with various biomolecules can enhance their biocompatibility, making them suitable for applications in drug delivery and biosensing. Overall, the polyol method offers significant advantages for producing Ag NPs with tailored properties for a range of biomedical applications [39, 40]. For example, Wiley et al. synthesized Ag NPs in which a syringe pump is used to inject two 3 mL solutions having different concentrations of AgNO_3_ and PVP at a specific rate into a very hot EG solution on continuous stirring. Several color changes were observed from yellow to brown, and it became stable after 46 h. Samples were washed with acetone and water to remove unwanted PVP and EG with gradual centrifugation at 16,000 rpm from 10 min to 1 h which was further characterized by SEM, TEM, and UV-vis spectrophotometry [41].

### 3.4. Light-Mediated Synthesis

In this modern technique, Ag NPs are prepared by their exposure to light [42]. Reduction is achieved by a source of light, for instance, laser radiation in the presence of stabilizing agents to control the size and shape of respective nanoparticles. Researchers have shown great success in creating fine, anticipated, and appropriately regulated Ag NPs. The constant light source melts silver nanospheres into silver nanoplates by altering their orientation. This phenomenon is termed as tailoring with light [43]. Ahmed et al. prepared Ag NPs using a mixture of ferredoxin–NADP^+^ reductase (FNR) and ferredoxin (FD) solution alongside AgNO_3_ salt. Exposing this aqueous solution to direct sunlight leads to the formation of Ag NPs within minutes showing a pale-yellow color that eventually leads to brownish color in 150 min indicating the formation of Ag NPs [44]. Similarly, Khamhaengpol and Siri prepared Ag NPs from the mixture of protein extract of weaver ant larvae, AgNO_3_, distilled water, glucose, and reducing sugar. This aqueous mixture was exposed to the fluorescent lamp for 6–72 h at room temperature to synthesize Ag NPs, which were continuously monitored via spectrophotometer at the wavelength range of 300–900 nm [45]. Recently, Rehman cosynthesized the Ag NPs by mixing an aqueous AgNO_3_ solution (1.25 mM) with extracellular polymeric substances (EPS) from *Chlamydomonas reinhardtii,* followed by exposure to light for 24 h. This light exposure facilitates the photochemical reduction of Ag^+^ ions to Ag NPs, as indicated by the emergence of a SPR peak between 425 and 480 nm in UV-vis spectroscopy. Control samples kept in the dark and EPS-only samples demonstrated that both light and silver ions are necessary for Ag NP formation, confirming the role of EPS in the synthesis process [46]. This year, Monalisha et al. synthesized the Ag NPs using an aqueous extract of seeds by optimizing the concentration of the extract, with 5 mL of various concentrations mixed with 20 mL of 1 mM silver nitrate (AgNO_3_) and incubated at 25°C in ambient light for 4 h. The rate of nanoparticle formation was monitored through UV-vis spectral analysis at regular intervals using a Shimadzu UV-1900i spectrophotometer. To assess the effect of white light intensity, sets of the optimal extract concentration were exposed to 250, 825, and 2000 lumens from LED bulbs, with one set kept in the dark for comparison. Following a 30-min incubation, one set was moved to the dark, while the other continued under the light for an additional 30 min. Final spectral analysis at 60 min enabled a comparison of the effects of light intensity on Ag NP synthesis, which is analyzed using software [47].

### 3.5. Seed-Mediated Synthesis

Seed-mediated synthesis is a fascinating method to produce desirable Ag NPs of different shapes and sizes [48]. The results obtained from this synthetic approach are greater as it utilizes nanomaterials as seeds for the controlled morphology of Ag NPs. Xin et al. prepared Ag NPs from gold seeds by mixing NaBH_4_ into a mixture of CTAB and HAuCl_4_.3H_2_O solution with continuous stirring yielding a pattern of colors from colorless to brownish yellow. The synthesized seeds had a size of 3 nm as determined by TEM and were subsequently used as precursors for the preparation of Ag NPs. The Ag NPs were synthesized by mixing different concentrations of seed solutions with AgNO_3_, reducing agent (NH_2_OH.HCl and NaOH), and CTAB solution which showed color change from light to dark red depending upon the seed concentrations within 30 min [49]. Wan et al. prepared seed-mediated Ag NPs by following the Lee–Meisel method in which different concentrations of citrate solution were added and boiled with deionized water into three-necked round bottom flasks equipped with a reflux condenser. Additionally, a mixture of seed solution and AgNO_3_ were added which were kept on vigorous stirring for 1 h on reflux and then cooled to room temperature. The synthesized Ag NPs were yellow-brownish in color and smaller ranging in size from 25 to 100 nm [50]. In another study, Ahmad et al. [4] synthesized Ag NPs following the method outlined by Faisal et al. Initially, a flask was filled with 90 mL of a 1 mM silver nitrate (AgNO_3_) solution and 10 mL of crude *Lemna* minor extract. To protect the sensitive reduced silver ions from light exposure, the flask was covered with aluminum foil. The mixture was then heated to 60°C for 45 min using a magnetic stirrer. After this, the solution was kept in complete darkness for 24 h to facilitate proper reduction. The color of the solution transitioned from pale brown to dark brown, indicating successful nanoparticle formation. Following this reduction, the mixture was centrifuged at 12,000 rpm for 20 min to isolate the pure Ag NPs. The resulting pellets were washed three times with distilled water and then dried in an oven at 80°C. Once dried, the material was mixed with a few drops of ethanol, ground into a powder, and stored at 7°C. Finally, the Ag NPs were formulated into a dust powder containing 1% Ag NPs as the active ingredient and 99% talc powder as the carrier. Recently, Habeeba and Raghavendra [51] synthesized the Ag NPs by mixing 5 mL of areca nut seed extract (ASE) with 25 mL of a 1 mM silver nitrate (AgNO_3_) solution while stirring continuously. The color change from red to dark brown within 90 s indicated the successful formation of ASE-Ag NPs. The reduction of silver ions (Ag^+^) to metallic silver (Ag^0^) is influenced by factors such as the concentration of the plant extract, reaction time, pH, and temperature of the solution. Consequently, ASE-Ag NPs were prepared using four different volumes of ASE (1, 2, 3, and 4 mL). The color development of the Ag NPs was observed at intervals of 15, 30, 45, and 60 min. Additionally, the impact of varying solution temperatures (303 K, 313 K, 323 K, and 333 K) and pH levels (5, 6, 8, 9, and 10) on ASE-Ag NP formation was examined, with the pH adjusted using 1 N NaOH and standardized with 1 N oxalic acid (COOH)_2_ [51]. Various synthetic procedures involved in the preparation of Ag NPs are demonstrated in Figure 1.

### 3.6. Electrochemical Synthesis

An extensive review of the literature highlights the application of electrochemical techniques for synthesizing Ag NPs, utilizing the foundational principles of electrolysis [52, 53]. For instance, Singaravelan and Bangaru Sudarsan Alwar detailed the electrochemical synthesis of Ag NPs using electrochemical deposition, a commonly employed method for producing metal nanoparticles. In this process, deposition occurs at the interface between an electrolyte solution containing silver ions and a conductive metal substrate. Specifically, Ag NPs were created using an electrolyte with a concentration of 0.01 mM AgNO_3_, prepared with double deionized water in a 100 mL cell. A glassy carbon electrode served as the working electrode, while silver metal acted as the counter electrode. The resulting Ag NPs exhibited dendritic growth, and it was observed that higher concentrations of silver ions promote aggregation and dendritic structures [54]. The reduction of silver ions at room temperature can be summarized as follows:

(1) $$\begin{array}{c} {\text{Ag}}^{+}+e^{-}⟶\text{Ag}. \end{array}$$

Furthermore, Theivasanthi and Alagar prepared Ag NPs following the electrochemical method with two graphite electrodes immersed in an electrolytic assembly containing a homogeneous solution of AgNO_3_ solution. A constant current supply results in the accumulation of Ag NPs on the surface of cathode, which was carefully collected and the nanopowder was obtained for further characterization [55]. Lim et al. synthesized Ag NPs using the electrochemical method by using AgNO_3_, EG, PVP, and potassium nitrate (KNO_3_) as a secondary electrolyte. The study employed the use of cyclic voltammograms containing platinum or boron-dipped diamond (BDD) electrodes to synthesize respective Ag NPs [56].

### 3.7. Microemulsion Synthesis

It is a typical method that is used for the synthesis of Ag NPs in which a metal ion solution is mixed with a reducing agent in two different phases that cannot be blended primarily [57, 58]. However, the mixing is achieved by using quaternary ammonium alkali salt. The surface of Ag NPs is shielded by a stabilizing agent which is nonpolar, thus remaining inactive in the organic medium. However, there are several drawbacks of using organic solvents for the synthesis which can cause various hazards. The final product must have only one solvent with little trace amounts of stabilizers or surfactants by successful partitioning. Hossain et al. prepared Ag NPs following the microemulsion method by preparing two clear water–oil emulsions consisting of SDS, cyclohexane, 1-pentanol, and water with metal precursor AgNO_3_ solubilized in one emulsion and reducing agent NaBH_4_ into another in equal amounts. The mixing leads to the appearance of a yellow color which indicates the formation of Ag NPs [59]. Sosa et al. prepared Ag NPs by following a precipitation reaction in a jacketed glass reactor with a reflux condenser and inlet attached to it. The reaction was initiated by inserting a microemulsion comprising AgNO_3_, surfactant, and toluene of which the temperature is raised to 70°C. A dropwise NaBH_4_ was added into the microemulsion followed by the addition of acetone to precipitate the product which was washed multiple times with acetone/water and then dried [60].

### 3.8. Microwave-Assisted Synthesis

Microwave-assisted synthesis is regarded as a safe method for the synthesis of Ag NPs as it constantly provides high amounts of Ag NPs with desirable properties in comparison with oil-mediated synthesis [61]. This method offers several advantages, including high production efficiency, minimal processing time, reduced safety risks, and lower aggregation of Ag NPs. Pal et al. prepared Ag NPs using microwave-assisted synthesis by using a mixture of ethanol, PVP, and AgNO_3_. The mixture was kept in the microwave for better kinetics and temperature stability for 5 s. The colorless solution turned to pale yellow instantly indicating the formation of Ag NPs [62]. Zhao et al. prepared Ag NPs by dissolving sodium alginate, AgNO_3_ in distilled water within a pH range of 5–11. The solution was kept in a microwave for heating in which the colorless solution turns to light brown indicating the formation of Ag NPs [63]. Various synthetic procedures including microwave-assisted synthesis involved in the preparation of Ag NPs are demonstrated in Figure 1.

### 3.9. Biological Synthesis

The biological synthesis of Ag NPs is an environmentally friendly and sustainable method that leverages the reducing and stabilizing capabilities of biological entities. This method, often referred to as “green synthesis,” offers a viable alternative to conventional chemical and physical approaches by eliminating the need for toxic reducing agents and high-energy inputs. The biological synthesis of Ag NPs involves the use of plant extracts, microorganisms (bacteria, fungi, and algae), and agricultural waste as reducing agents [23, 27]. The process typically involves mixing silver salt solutions (AgNO_3_) with the chosen biological extract, where bioactive compounds such as proteins, flavonoids, phenolic compounds, and enzymes facilitate the reduction of Ag^+^ ions to Ag^0^. The reaction parameters, including pH, temperature, and incubation time, significantly influence the nanoparticle's size and morphology [43].

The types of biological agents used [43, 46, 47, 64]:

• Plants: Various plant extracts, such as those from *Trigonella foenum-graecum* and *Ribes rubrum*, have been reported to facilitate Ag NP synthesis due to their high antioxidant content.
• Bacteria: Several bacterial species, including *Pseudomonas* and *Bacillus,* have been utilized for Ag NP synthesis, often secreting extracellular enzymes that mediate the reduction process.
• Fungi: Fungal-mediated synthesis offers advantages due to their large biomass production and secretion of reducing metabolites.
• Microalgae: Algal species, such as *Chlamydomonas reinhardtii*, have been explored for Ag NP synthesis through EPS.
• Agroindustrial Waste: Waste materials like fruit peels and seed extracts provide a cost-effective and sustainable route for Ag NPs production.

The biological synthesis of Ag NPs presents multiple benefits, including ecofriendliness, as it avoids toxic chemicals, making it safer for biomedical and environmental applications. It is also cost-effective due to the use of readily available biological resources, reducing overall synthesis costs [64]. Additionally, biocompatibility is a significant advantage, producing stable nanoparticles suitable for medical and antimicrobial applications. However, challenges remain, such as scalability issues, as variability in biological extracts can lead to inconsistent nanoparticle synthesis. The synthesis rate is also slower compared to chemical methods, requiring longer reaction times. Furthermore, complex purification steps are often needed to remove residual biomolecules from synthesized nanoparticles, adding to processing costs [43].

Recent studies have demonstrated the effectiveness of biological synthesis. For instance, white LED light-mediated photocatalysis has been used to enhance Ag NP synthesis from *Trigonella foenum-graecum* seed extracts [47]. Additionally, microbial-assisted synthesis using *Chlamydomonas reinhardtii* has been explored for its unique EPS-mediated reduction mechanism [46]. Moreover, agroindustrial waste-derived Ag NPs have been investigated for their potential applications in antimicrobial and antioxidant activities. The biological synthesis of Ag NPs is a promising and sustainable approach with diverse applications in medicine, environmental remediation, and material sciences [64, 65]. Despite some limitations, ongoing research continues to optimize synthesis parameters to improve yield, stability, and functionality.

## 4. Toxicity of Ag NPs

Toxicity of Ag NPs has been a major concern for researchers globally, and modern research has opted for various biochemical and physical methods to overcome this issue [66]. Studies have reported the deposition of silver in skin which leads to a condition called argyria [67]. However, the deposition of silver is not only limited to the skin but propagates to other organs which are the brain, kidney, liver, blood, small intestine, pancreas, teeth, muscles, heart, and salivary glands. It is reported that in renal absorption, silver is found in the glomerulus membrane, while in the brain, it is found in the hippocampus and pons. Studies have also reported the transfer of silver from mothers to offspring highlighting the possibility that silver can cross the placental barrier [68].

Research indicates that certain organs are more susceptible to silver deposition than others. Experimental studies have shown that orally administered silver accumulates more significantly in the intestines compared to the stomach, liver, kidneys, and pancreas. In contrast, only trace amounts are found in the blood, heart, lungs, and muscles. In the excretion phase, Ag NPs are also found in urine as an orally administered drug. Its impact on reproductive, cardiovascular, gastrointestinal, body weight, neuro, and immunotoxicity has been observed in various studies [69].

To counter this critical issue, researchers have adopted ways to remove excess silver from colloidal solutions [70]. Aggregation of Ag NPs in aqueous solvents is observed which is overcome by using ultrasonication. Ag NPs aggregates are ruptured by applying large amounts of energy through sonication to form smaller aggregates. As a result of this, the energy applied is transformed into heat energy which increases the temperature resulting in the Ag NPs becoming Ag ions. The heat generated by the sonication procedure can produce toxic batches so to prevent this, cooling is preferred after sonication of the Ag NPs sample to appear less toxic as compared to the samples that were kept without cooling [71].

Apart from ultrasonication, various methods can help minimize the toxicity of Ag NPs. One convenient approach is ultracentrifugation, which separates Ag NP pellets from the surrounding medium, effectively removing excess silver ions. Continuous washing and redispersion of the Ag NP pellets in fresh solvent further eliminates residual silver ions, thereby reducing toxicity [72].

## 5. Protein-Based Nanoparticles

### 5.1. Overview

Nanotechnology has emerged as one of the best modern methods for novel drug delivery systems because the methods utilized during preparation are cost-effective, nonhazardous, and convenient [73]. The materials used in the synthesis are natural polymers that are readily available, biodegradable, biocompatible, and economical while being present abundantly in nature. Researchers are now focused on developing nanocarriers that can transport drugs conveniently, readily absorb, and excrete without causing any harmful effects. Nanotechnology has given an insight into the use of proteins as nanocarriers as they are regarded as generally regarded as safe (GRAS) because of their inducible properties that include non-antigenicity, high binding affinity, increased nutritive importance, and therapeutic benefits [74]. The surface chemistry of proteins is highly adaptable as it can adhere to several functional groups, which can interact with different biomolecules, and can form three-dimensional complex structures—offering interactions such as cross-linking or encapsulating them into the matrix [75].  Table 1 represents different proteins used for the preparation of nanoparticles.

### 5.2. Factors That Affect Protein Nanoparticle Preparation

#### 5.2.1. Composition

**Figure 2:**
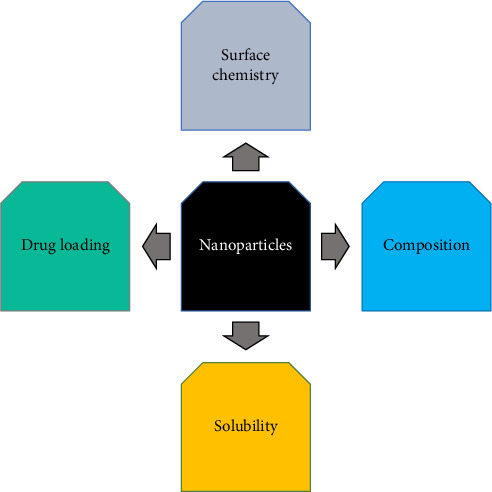
Factors which can affect the preparation of nanoparticles that include (i) composition, (ii) solubility, (iii) surface chemistry, and (iv) drug loading.

The synthesis of nanoparticles is highly dependent on the stoichiometry of the protein and its source. Proteins have different molecular weights according to their amino acid sequence; therefore, any changes in these sequences can have a direct effect on the nanoparticle properties, for example, human serum albumin (HSA) contains a thiol group that is highly reactive and can form dimers with other biomolecules. Formation of these dimers can lead to polydispersity and form large aggregates in the nanoparticle's solution [9]. As reviewed in the literature, researchers are now focusing on the controlled-sized synthesis of protein-based nanoparticles by adding a surfactant or a capping agent. This is achieved by using different surfactants which include PVP, polyvinyl alcohol (PVA), and polyethylene glycol (PEG) to compensate for this composition behavior. In contrast to this, plant proteins contain amino acids having different molecular weights and pigments that might affect their preparation. Considering this, the protein must be purified before preparing nanoparticles to avoid interference in the form of aggregates [97].  Figure 2 shows the factors that can affect the preparation of protein-based nanoparticles.

#### 5.2.2. Solubility

The solubility of protein is a critical parameter for nanoparticle preparation as it largely depends on the choice of method and the solubility characteristics of the protein. Proteins are soluble in different mediums because of their surface charge and different functional groups present on their exterior surface. The solubility characteristics are largely dependent on the polar properties of the solvent. The solubility characteristics are largely dependent upon the isoelectric point (pI) of the given protein and the pH of the dissolving solution [98].

#### 5.2.3. Surface Chemistry

The external surface of proteins contains many functional groups which include carboxyl, amine, and thiol, which play an important role in the surface properties of nanoparticles. By modifying these chemical functional groups, many essential properties can be introduced in the final resultant nanoparticles which include drug loading, biocompatibility, distribution, in vivo stability, etc. Moreover, cross-linkers such as glutaraldehyde and stabilizing agents such as PVP, PVA, and PEG are used to maintain its structure, prevent phagocytic behavior within the body, and carry out reactions with desired results. Additionally, drug loading can be achieved via electrostatic attractions to enhance the binding affinity between the drug and protein molecule [99, 100].

#### 5.2.4. Drug Loading

The loading of a drug within the nanoparticles follows different pathways such as adsorption or encapsulation with the assistance of covalent, noncovalent, electrostatic, and hydrophobic interactions. Several factors have been known to affect the nanoparticle's binding and release which include diversity in molecular weight, solubility properties, and log *P* characteristics which affect the release of a drug from the nanoparticles [101, 102]. The factors that influence the physicochemical characteristics of nanoparticles are illustrated in Figure 2.

### 5.3. Preparation Methods

**Figure 3:**
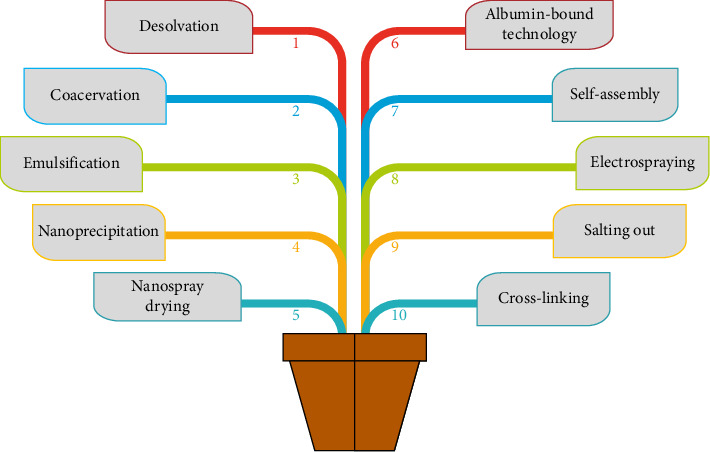
Graphical illustration of various methods utilized in the preparation of PBNPs.

The preparation of protein-based nanoparticles requires optimum conditions because slight deviations in these parameters (pH, temperature, and choice of solvent) can lead to loss of protein structure [103]. However, the formation of nanoparticles is dependent upon the decrease in hydrophobic interactions and in the increased rate of unfolding of protein structure. The protein goes through conformational changes based on its concentration, composition, pH, solvent, ionic strength, and different methods to form the respective nanoparticles [9]. Various methods for the preparation of protein-based Ag NPs are demonstrated in Figure 3.

#### 5.3.1. Desolvation

This method is based on the addition of a desolvating agent into an aqueous solution of protein, leading to dehydration of its structure from stretched to coil conformation. Galiyeva et al. prepared an aqueous solution of BSA in which a desolvating agent is added dropwise such as ethanol. The appearance of turbidity in the solution indicates the formation of BSA nanoparticles, resulting from aggregation induced by the desolvation process. The study showed the addition of L-cysteine/urea instead of a synthetic cross-linker such as glutaraldehyde to provide stability. The characterization of synthesized nanoparticles was carried out by using different techniques to ensure the successful formation of protein nanoparticles [104]. Pandey et al. prepared lactoferrin (Lf) nanoparticles using the desolvation method in which Lf was dissolved in saline on continuous stirring at a heated temperature. The pH of the solution was kept alkaline using NaOH. The induction of organic solvents (ethanol, acetone, and isopropanol) leads to the turbid appearance of the solution indicating the formation of nanoparticles. Glutaraldehyde was added to ensure successful cross-linking of the synthesized nanoparticles which is further characterized using several techniques [105].

#### 5.3.2. Coacervation

The coacervation method is based on the modulation of protein–solvent interactions, where changes in parameters such as pH, ionic strength, electrolyte concentration, and solvent polarity alter the net charge and hydration shell of protein molecules. These variations reduce protein solubility by diminishing electrostatic repulsion and promoting intermolecular interactions, ultimately leading to liquid–liquid phase separation and the formation of a protein-rich coacervate phase [9]. Krishna Sailaja et al. prepared protein nanoparticles (PBNs) by the addition of ethanol into an aqueous solution of BSA until turbidity was observed. Subsequently, glutaraldehyde and Tween 20 to ensure cross-linking of all amino acid residues, followed by ethanolamine was added to block the unreacted aldehyde group and to stabilize nanoparticles. Large aggregates were centrifuged and ultrafiltered through acetate membrane. Characterization was carried out using multiple biochemical techniques to carry out analysis of synthesized PBNs [106]. Lin et al. prepared Chitosan–EDTA Ag nanocomposites using the coacervation method by making a solution of Chitosan–EDTA in deionized water on heating. Dropwise addition of ethanol leads to a change in color which was further cross-linked by using glutaraldehyde. The resulting nanoparticles were centrifuged and resuspended into ethanol. AgNO_3_ solution was added further into the nanosuspension of the Chitosan–EDTA complex which was left on stirring for 10 min at room temperature. The reaction was further dialyzed for the removal of EDTA molecules to ensure the successful capping of Ag ions onto Chitosan nanocomposites [107].

#### 5.3.3. Emulsification

This method uses extensive homogenization/ultrasonication, followed by evaporation of solvent during continuous magnetic stirring at room temperature or under reduced pressure [108]. The method is dependent upon a single emulsion (water in oil) or either double emulsion (water-in-oil-in-water). Mishra et al. prepared albumin PEGylated nanoparticles by the dropwise addition of 100 mL of olive oil on continuous stirring for 30 min into an aqueous solution of MAL-PEG-BSA solution which was further sonicated for 5 min to create a primary emulsion. Another secondary emulsion of glutaraldehyde and toluene was prepared which was sonicated to produce a secondary emulsion. The secondary emulsion was then added into the primary emulsion on constant stirring for 2 h which was further collected by ultracentrifugation. The resultant particles were washed, dried, and freeze-dried for further analysis [109]. Zhao et al. prepared gelatin nanoparticles by dissolving gelatin in deionized water which was slightly warmed to 30°C. The gelatin solution was added dropwise into a solution of poloxamer to form an emulsion. 10% glutaraldehyde was added to ensure successful cross-linking within emulsion which was cooled to 5°C in an ice bath. Characterization of gelatin nanoparticles was carried out using several biochemical techniques to confirm the synthesis of PBNs [110].

#### 5.3.4. Nanoprecipitation

Nanoprecipitation occurs by rapid desolvation of protein when the protein solution is added to the nonsolvent. Precipitation of protein occurs as the protein disperses into the diffusing medium [111]. Tarhini et al. prepared albumin nanoparticles using the nanoprecipitation method by preparing different concentrations of BSA solutions which were precipitated out using a desolvating agent such as ethanol and a cross-linking agent such as glutaraldehyde. Effects of BSA concentrations, ionic strength, Ethanol injection rate, different pH values, and process variation were determined to calculate nanoparticle yield. Different biochemical techniques were utilized to carry out characterization to visualize and determine the synthesized uniform PBNs [112]. Zeng et al. synthesized insulin nanoparticles using the flash nanoprecipitation (FNP) method by preparing an aqueous solution of insulin and ionic surfactants in dichloromethane with assistance from ethanol to form a liquid mixture. The addition of water into this liquid mixture leads to the partitioning of insulin molecules into the organic phase resulting in FNP which were further labeled by fluorescein isothiocyanate (FITC) for quantification and detection [113].

#### 5.3.5. Nanospray Drying

Spray drying is an efficient technique for transforming liquids into fine particles through a continuous process, particularly suited for preserving the activity of heat-sensitive molecules like proteins. The process unfolds in four key stages: atomization, contact with drying air, drying of the atomized spray, and final separation of the dried product from the drying gas. PBNPs generated via spray drying can be optimized using the Taguchi method, which assesses factors like spray mesh size, nitrogen flow rate, inlet temperature, and protein–surfactant concentrations. For instance, using a Nanospray Dryer B90, researchers dissolved bovine serum albumin (BSA) with Tween-80 (as a surfactant), filtered it, and sprayed it under various conditions, with dried particles collected for further characterization [114]. The spray-dried protein-based nanoparticles are prepared via the Taguchi method which is based on (1) spray mesh size, (2) nitrogen flow rate, (3) inlet temperature, and (4) protein and surfactant concentration. A Nanospray Dryer B90 is used in which BSA and Tween-80 (surfactant) are dissolved and filtered through syringe filters which were sprayed at various experimental conditions. Dried powder is obtained from a particle scraper of an electrode and stored for further characterization [115]. Kim et al. prepared Ag NPs by adding AgNO_3_ solution into an aqueous solution of PVP by employing the nano spray drying method using a Nanospray Dryer B290 [116].

#### 5.3.6. Albumin-Bound Technology

An effective and safe technique in nanotechnology involves the encapsulation of poorly water-soluble drugs. Albumin serves as a carrier molecule, enabling the encapsulation of drugs such as traditional taxemes, nab-paclitaxel, and curcumin, among others [104]. High water-solubility nanoparticles containing paclitaxel and curcumin are formulated using HSA via nanoparticle albumin-bound (NAB) technology, involving chloroform presaturated with water [117]. Lee et al. prepared PEG-HSA nanoconjugates by dissolving PEG in PBS buffer on constant stirring. A solution of HSA was added to the above mixture at room temperature, and the stirring was continued till 12 h. The resulting nanoconjugates were dialyzed, lyophilized, and stored at freezing temperature for further analysis to ensure the successful synthesis of nanoconjugates [118].

#### 5.3.7. Self-Assembly

Hydrophilic proteins like albumin can be modified into hydrophobic proteins in the form of protein micelles when it is inducted into an aqueous solution [118]. The hydrophobic core of proteins can serve as a suitable binding site for various drugs. To enhance the lipophilicity of nanomicelles, octyl-modified BSA nanoparticles were synthesized. Subsequently, methoxy polyethylene glycol (mPEG) was conjugated to plant protein zein to create self-assembling nanomicelles capable of encapsulating paclitaxel, which was loaded using octyl-modified BSA. This approach resulted in increased solubility and stability of the drug during cellular uptake [119]. Xie et al. prepared lysine nanoparticles using the self-assembly method. The self-assembly of PBNs was managed by PEG which modifies the surface of PBNs by the formation of covalent bonds with thiol functional groups present on the exterior surface [120].

#### 5.3.8. Electrospraying

Electrospraying methods are used to synthesize PBNs in which a protein–drug solution is formed in a capillary which is pulled out by applying a high current voltage forming a thin jet [121]. The formation of nanoparticles depends upon the applied voltage and the nature of protein solutions. Zein nanoparticles loaded with docosahexaenoic acid are prepared and visualized under the optical microscope which displays fluorescent images of zein nanoparticles. The encapsulated docosahexaenoic acid showed the same results in comparison with free zein nanoparticles with controlled morphology indicating effective encapsulation within the nanoparticle [122]. Wu et al. prepared elastin-like polypeptide (ELP) nanoparticles by using the electrospraying method in which an aqueous solution of ELPs was filled in a syringe which was dispensed at a specific flow rate. An electric field was generated using a power supply of high voltage between the collector and nozzle. The electrospraying of ELP solution was carried out by using a silica nozzle of a specific diameter in a vacuum chamber or an inert gas to avoid loss of electrical charge. The formation of a cylindrical electrical field helps in the atomization of ELP nanoparticles which was further characterized by field emission scanning electron microscope (FESEM) [86].

#### 5.3.9. Salting Out

This technique is based on the separation of solvent from aqueous solution which minimizes stress to protein carriers because it does not include temperature modification which is suitable for heat-sensitive molecules [123]. Drawbacks include costly washing steps and limited to lipophilic drugs. Protein and drug are dissolved in the same solvent normally acetone which is emulsified in aqueous gel containing the salting-out agent. Salting-out agents used for the preparation of protein-based nanoparticles along with stabilizers generally are electrolytes which are magnesium chloride, calcium chloride, magnesium acetate, etc. A dilution step with an aqueous solution is done to enhance the diffusion of acetone into an aqueous phase which leads to the formation of nanoparticles. The salting-out agent and solvent are eliminated via cross-flow filtration [124]. Teng et al. prepared Cabazitaxel–HSA nanoparticles using the salting-out method by forming a mixture of sodium dihydrogen phosphate and ethyl alcohol containing Cabazitaxel at vigorous stirring. A solution of HSA was injected into this mixture at 65°C for the formation of nanoparticles followed by a rapid decrease in temperature to 8°C. Ultrafiltration was performed to remove the excess ethanol and unbound Cabazitaxel which was followed by freeze-drying of the sample [125].

#### 5.3.10. Cross-linking

Cross-linking methods are used to increase the stability of protein and to enhance sustained drug delivery. Various types of cross-linkers are used to prepare protein-based nanoparticles which include chemical, ionic, thermal, and enzymatic; however, they can affect the particle stability if not removed properly and can induce toxicity to biological systems [126]. HSA nanoparticles were prepared in the presence of glutaraldehyde as a cross-linker to study its effect on the size and isoelectric point by preparing different glutaraldehyde concentrations. Various concentrations were prepared based on the amount of cross-linker required to cross-link all lysine residues in the matrix. The resulting product showed that cross-linkers do not affect particle size; however, pI decreases as glutaraldehyde concentration increases [127]. Similarly, researchers have designed BSA nanoparticles using light as a cross-linking agent by dissolving freeze-dried BSA into PBS followed by the addition of tris-bipyridyl ruthenium and ammonium persulfate into the resulting solvent. The solution was exposed to visible light for cross-linking using an incandescent lamp, while it was inhibited by using dithiothreitol after required exposure. The sample was further preceded for ultrafiltration against PBS and characterized for analysis [102].

## 6. Protein-Based Ag NPs

Proteins are excellent biological macromolecules that possess significant importance in developing applications in the fields of theranostics, therapeutics, and biomedicine. Researchers have chosen proteins over other biomolecules because of their structural modifications, biodegradable nature, bioavailability, and lower toxicity [128]. As a result, protein-based nanoparticles have become a promising candidate for targeted drug delivery in vivo. However, to increase the efficacy of protein-based nanomaterials, researchers have opted for various peptide–nanoparticle conjugates with the peptide molecules. Bioconjugation has sparked great attention in the field of nanomaterials as it will offer the advantages of displaying active regions of peptides and providing insights for effective diagnosis and therapy for cancer treatment [129]. Moreover, conjugating peptides with metal ions will develop detection systems because of their ability to absorb light and heat with added applications of killing tumor cells, bacteria, and fungi. Bioconjugation can be achieved either by electrostatic attractions, direct contact, covalent attractions, and secondary interactions. Given the interesting facts stated above, the metal-conjugated PBNs provide innovativeness and an excellent option to tailor with the stoichiometry of peptide molecules—offering greater stability and decreasing their toxicity within cells [130].

Ag NPs stabilized with proteins represent an emerging field due to their unique properties and versatility in biomedical and technological applications. These nanoparticles, typically ranging from 10 to 100 nm, adopt spherical or prismatic forms controlled by the protein used as a stabilizing agent. Protein not only prevents aggregation but also enhances biocompatibility and improves functionality by serving as a coating that stabilizes the particles and reduces their toxicity to living organisms. Additionally, the surface of the Ag NPs can be modified by the functional groups of the proteins, which enhances their ability to interact with biomolecules and biological systems, making them suitable for applications in antimicrobial therapies, cancer treatments, and controlled drug delivery systems [131]. Below are some examples of silver-conjugated PBNs which are as follows:

### 6.1. Albumin

Albumins belong to the family of globular proteins found abundantly in egg white and blood plasma. It is a water-soluble protein that denatures upon successive heating. As a promising drug delivery candidate, albumin has sparked great interest among researchers in forming nanoconjugates with metal ions. Majeed et al. prepared albumin-capped Ag NPs with a slightly modified method. The 1000 μL of BSA (1% W/V) was added on continuous stirring into a solution of AgNO_3_, and color changes were observed which was then proceeded to orbital shaking at 120 rpm for 12 h [132]. Pant et al. prepared albumin-capped silver nanomaterials using a slightly modified method provided by [133]. AgNO_3_ solution was stirred on a hot plate, and citrate solution was added dropwise. The color change was observed indicating the successful formation of Ag NPs having a size of 62 nm which was confirmed by dynamic light scattering (DLS), UV, and fluorescence spectroscopy [134]. Yang et al. synthesized BSA-capped Ag nanoclusters by combining 5 mL of BSA and AgNO_3_ solutions in the dark under continuous stirring. Subsequently, a freshly prepared NaBH_4_ solution was added to the mixture, resulting in a color change from pale to brown. The reaction mixture was stirred for an additional 4 h to optimize the synthesis of BSA-Ag nanoclusters, which was confirmed through fluorescence spectroscopy [135]. BSA-capped Ag nanoclusters were prepared using a two-step reduction process involving NaOH and NaBH_4_ as reducing agents. Initially, 250 mg of BSA was dissolved in deionized water, followed by the addition of 0.1 M AgNO_3_ at ambient temperature. After a 5-min incubation period, NaOH was added, and 1 h later, NaBH_4_ was introduced while the solution was continuously stirred. The resulting solution was further purified through dialysis and freeze-dried to maintain the stability of the BSA-capped Ag nanoclusters [136].

### 6.2. Gelatin

Gelatin is an odorless, colorless, translucent protein that is obtained from collagen. It is mostly found in bones and cartilages of animals, and because of its sticky gummy nature, it is used as an additive in food products and medications. Researchers have used gelatin–metal ion nanoconjugates because of their biocompatibility and lower toxicity in vivo [137]. Kanmani and Rhim prepared gelatin–Ag nanocomposites by adding a boiling solution of AgNO_3_ of various concentrations dropwise into an aqueous gelatin solution having sorbitol. The pH of the solution was kept alkaline at pH 8 using NaOH. The color change was observed from white to yellow, while the solution was transferred to a glass Teflon plate for drying at room temperature [138]. Halder et al. synthesized gelatin–Ag nanocomposites using different protein concentrations against AgNO_3_ using a chemical reduction method. Aqueous solutions of gelatin and AgNO_3_ were mixed and gently stirred for 30 min at room temperature. A freshly prepared NaBH_4_ solution was added to reduce the silver ions which were observed via color change from colorless to golden brown. The solution was kept on continuous stirring to ensure successful synthesis of nanocomposites which was confirmed by UV spectroscopy, particle size, and TEM [139]. Pourjavadi and Soleyman synthesized gelatin–Ag nanoshells using a light-mediated green synthesis method by dissolving and homogenizing gelatin in water at a moderate temperature. The addition of AgNO_3_ into the aqueous solution of gelatin was exposed to UV sunlight which after 2 h changes color to orange-brown. The samples were further purified using dialysis to remove any excess silver ions from the solution [140]. Loan Khanh et al. prepared gelatin-stabilized silver nanocomposites (SNCs) by dissolving gelatin in deionized water under continuous stirring. The addition of AgNO_3_ into the gelatin solution was further proceeded with exposure to UV light for 6 h, leading to the formation of gelatin–SNCs. Particle size was determined, and quantitative analysis of gelatin–Ag NPs was done by using atomic absorption spectroscopy [141]. Darroudi et al. prepared gelatin Ag NPs in a UV reactor by exposing gelatin–Ag NPs to UV light at different times. The synthetic method involves dissolving gelatin in deionized water on stirring in which AgNO_3_ was added and subsequently irradiated with UV light under a UV reactor. The synthesis was confirmed and characterized using UV-vis spectroscopy, TEM, XRD, and AFM analysis [142].

### 6.3. Casein

Casein belongs to the family of phosphoproteins and is abundantly present in mammalian milk. The major uses of casein involve additives in food and dairy products. Due to its ease of availability, casein is an ideal candidate for bioconjugation with metal ions to form respective nanoparticles [143]. Ashraf et al. prepared casein–Ag NPs by preparing an aqueous solution of 1% casein and dissolving it in 10 mL of Tris buffer in a strongly alkaline environment on continuous stirring. The solution of AgNO_3_ was boiled, and the casein suspension was inducted into the boiling solution of AgNO_3_. Several color changes were observed from transparent to yellow-brown indicating the formation of Ag NPs. The reaction mixture was further preceded by purification through centrifugation and was stored at room temperature for further experimentation [144]. Selvaraj et al. prepared casein–Ag NPs using the chemical reduction method under cold experimental conditions by the addition of AgNO_3_ solution into a solution of NaBH_4_ dissolved in 5% SDS on gentle stirring. A 5% casein solution with polyethylene oxide (PEO) was further added to form Ag NPs which was confirmed by characterization techniques [145]. Ghodake et al. prepared casein–Ag NPs using a highly reproducible green synthesis method by using casein hydrolytic peptides (CHP), NaOH, and AgNO_3_ of various concentrations and volumes. The final volume of the resultant mixture was 20 mL, and the reaction was carried out and maintained at 60°C [146]. Tavaf et al. prepared casein–Ag NPs with comparison to control by forming a mixture of whole casein fraction (WCF), AgNO_3_ in deionized water, and NaOH. The reaction mixture was maintained at 37°C for 2 days in which color changes were observed eventually leading to brown from pale yellow. The synthesis was confirmed by using FTIR, UV-vis spectroscopy, and DLS [147].

### 6.4. Collagen

Collagen is widely distributed among connective tissues and is an abundant component of skin, blood, bones, and hair. Because of its diverse applications in wound healing, surgical procedures, and scar formation, researchers have conjugated metal ions with collagen to form metal–collagen nanostructures [148]. Craciunescu et al. prepared collagen–Ag NPs by adding a solution of various concentrations of AgNO_3_ dropwise in 0.3% collagen gel at pH 5.5. The mixture was robustly shaken and incubated for 60 min at room temperature [149]. Cardoso et al. prepared collagen-based Ag NPs using a chemical reduction method by mixing an aqueous solution of AgNO_3_ and collagen in equal volumes at agitation. The reducing agent, that is, NaBH_4_ was added to the following mixture in the form of the jet which was homogenized and centrifuged to remove excess silver ions from the solution [81]. Patrascu et al. synthesized collagen scaffolds based on Ag NPs at pH 9 by neutralizing Ca(OH)_2_ with collagen gel containing silver ions for 24 h. Na_2_HPO_4_ interacted further with this collagen gel which was incubated for more than 24 h to nucleate hydroxyapatite (HA) from collagen. The resultant product is cross-linked and freeze-dried for further analysis [150]. Nogueira et al. synthesized collagen-based Ag NPs using a chemical reduction method in the presence of natural stabilizers. The solutions of collagen and AgNO_3_ were mixed and homogenized with each other followed by the addition of NaBH_4_ for the reduction of silver ions. Natural polymers such as cashew gum, agar gum, and K-carrageenan gum were added to stabilize the synthesized Ag NPs [151]. Deoxygenation of AgNO_3_ in aqueous solution was performed for 30 min using an N_2_ purge which is followed by the dropwise addition of collagen into the reaction mixture. The final resultant product mixture was purged and irradiated on a photoreactor [152].

### 6.5. Immunoglobulins (IGs)

IGs, commonly known as antibodies, are a critical part of our immune system that targets antigens that have invaded our immune system. Due to their selective targeting nature, IGs conjugated with metal ions in the state of nanoparticles provide an ideal basis for targeted drug delivery, binding with antigens, recognizing bacterial cells, and killing them [153]. Matea et al. synthesized IgG-based SNCs by the addition of sodium citrate into the boiling solution of AgNO_3_ on constant stirring. The color change was observed from transparent to pale yellow, indicating the formation of Ag NPs. Immunoglobulin G (IgG) was bonded with calcium folinate with assistance from N-hydroxy succinimide (NHS) and 1-ethyl-3-(3-dimethylaminopropyl) carbodiimide (EDC) that helps to cross-link protein molecules with calcium folinate (Cf). The purification was achieved using successive centrifugation and resuspension in deionized water to remove any excessive cross-linking agents [154]. Batistela et al. synthesized human IgG-functionalized Ag NPs having a size of less than 10 nm by using the chemical reduction method. Dissolution was carried out using equal volumes of AgNO_3_ and sodium citrate into deionized water which was further proceeded by the addition of NaBH_4_. The binding of IgG with Ag NPs colloids was achieved through mercaptosuccinic acid (MSA) which was kept at 50°C for 150 min followed by the addition of phosphate buffer and anti-IgG to carry out aggregation immunoassay [155]. Lizoń et al. synthesized an Ag NPs-based assay to develop a detection system of IG light chains by chemical reduction method by forming a mixture of AgNO_3_ and sodium citrate in ice-cold deionized water. Dropwise addition of NaBH_4_ on gentle stirring showed a color change to yellow coloration, and the coating was provided by the addition of anti-FLC Ig serum which was incubated at 37°C for 30 approximate minutes [156]. Paul et al. prepared Ag NPs tagged with IGs using a chemical reduction method by reducing AgNO_3_ with sodium citrate at pH 4.5. The reaction was carried out in the dark, and tetrahydrofuran (THF) was added to stabilize the nanoparticle formation. 1 mL of the stock solution of synthesized Ag NPs was added into 400 μL of anti-IgG solution which was further centrifuged thrice and the pellet was resuspended into deionized water [157].

### 6.6. Enzymes

Enzymes are biological catalysts that accelerate chemical kinetics in the body. Due to their abundance, these protein molecules offer greater surface modifications for metal ions and drugs to bind and provide an excellent platform for targeted drug delivery [158]. Mishra and Sardar synthesized α-amylase Ag NPs by using itself as the reducing agent. The Ag NPs formation was initiated by forming a suspension of α-amylase solution in Tris-HCl buffer having pH 8.0 and AgNO_3_ solution at 25°C [159]. Dumri and Hung Anh synthesized nanocomplexes of lipase–Ag NPs–polydopamine (LPA complex) by the addition of dopamine hydrochloride and lyophilized lipase suspended in phosphate buffer into AgNO_3_ solution dissolved in Tris buffer having pH 8.5. The complex was further purified using centrifugation and kept at 4 for further analysis. Thermal stability and characterization were carried out using SEM, TEM, and UV-vis spectroscopy [160]. Mirzajani et al. synthesized acetylcholinesterase (AChE) Ag NPs using a chemical reduction method. AgNO_3_ solution dissolved in PVA was reduced by the addition of sodium citrate under constant stirring at 96°C. The reaction mixture was cooled down to 4°C after successful synthesis and set aside in the dark for further analysis. The addition of AchE into the Ag NPs solution leads to a nanocomplex of AchE-Ag NPs which was confirmed by UV spectroscopy and DLS [161]. Meroliya et al. reported Ag NPs of lipase and 200 mL AgNO_3_ solution [162]. Baskar et al. prepared L–asparaginase–Ag NPs–FITC bio-nanocomposite by initially synthesizing Ag NPs using the citrate reduction method. FITC was added on continuous stirring at 30°C for 2 h. Asparaginase was added into this suspension on continuous stirring, which was then centrifuged, washed, and freeze-dried for further use [163].

### 6.7. Silk Proteins

Silk proteins are water soluble and belong to the class of glycoproteins. It comprises two proteins, namely, fibroin and sericin which are obtained from silkworms. Due to its self-assembly, slow biodegradability, and biocompatibility, it has become an exciting candidate for targeted drug delivery in conjugation with metal ions [164]. Shivananda et al. prepared silk fibroin (SF)–Ag NPs by using SF as a reducing and stabilizing agent. The synthetic procedure involves the addition of AgNO_3_ powder into a 10% SF solution to form a respective mixture [165]. Dhas et al. synthesized silver-coated SF nanoparticles for wound healing applications by using a green synthesis approach and a simple adsorption dipping method. The *ex situ* method involved the interaction of colloidal silver with silk fibers for 6 h while the in situ method involved the interaction of silk fibers with plant extract and AgNO_3_ for 6 h [166]. Aramwit et al. prepared silk sericin–Ag NPs in a highly alkaline medium using NaOH. The silk sericin solution was diluted in various concentrations and added on continuous stirring in a solution of AgNO_3_. The solution was kept overnight for a reduction on stirring. Color changes were observed from transparent to the formation of yellow colloidal Ag NPs ensuring the successful synthesis of Ag NPs [167]. Pei et al. synthesized Ag NPs–SF–carboxymethyl chitosan (CMC) sponge for wound healing by mixing a solution of AgNO_3_ and SF under UV light for 6 h. Different analytical techniques validated the synthesis of SF-Ag NPs which were later added to the CMC solution to form respective sponges after going through its synthetic process [168]. Calamak et al. prepared SF–Ag nanofibers using the electrospinning method. Initially, fibroin was dissolved in formic acid in which a solution of AgNO_3_ was added. The parameters were optimized required for electrospinning at a voltage of 17 kV and specified flow rate, and the resulting nanofibers were collected on a plate. Exposure to UV light was provided to SF–Ag nanofibers for half an hour to synthesize the respective nanofibers. The synthesized nanofibers were cross-linked with glutaraldehyde, kept in a desiccator and at ambient temperature [169].

## 7. Key Parameters for Evaluating Instrumental Techniques in Protein-Based Ag NPs

Various instrumental techniques are utilized for the physicochemical characterization of protein-based nanoparticles to determine the morphology, size, surface charge, and various other parameters. These techniques are critical for the successful physicochemical characterization of nanoparticles synthesized [170]. Various mathematical equations about the chemistry of nanoscale materials are considered to ensure product formation. These parameters include several analytical techniques which are listed as follows.

### 7.1. Photon Correlation Spectroscopy (PCS)

PCS is a laser-based technique that is used to measure nanosized particles. In this method, the time decay of the near particle caused by the Brownian motion helps to evaluate nanoparticle structure via the Stokes–Einstein equation as follows:

(2) $$\begin{array}{c} D=\frac{k_{B}T}{6\pi \eta r_{h}}. \end{array}$$

In the above equation, *D* represents the diffusion coefficient (m^2^/s) and *η* represents the dynamic viscosity of the fluid (Pa·s or N·s/m^2^). Likewise, k_B_ is the Boltzmann constant (1.38 × 10^−23^ J/K), T is the absolute temperature (Kelvin, K), and *r*_*h*_ is the hydrodynamic radius of the particle (meters, m). The major drawback of this technique is that it does not create a high-resolution histogram of the size distribution [171]. Despite all the drawbacks, PCS has been widely used as a characterization tool for the determination of silver NPs size. In one of the studies, PCS was performed after supplementing the 1.8 mL of colloidal silver suspension with 200 μL water, or with 200 μL of maltose binding protein (MBP−Ag4) or NaCl stock solutions to final concentrations of 5 μL. The concentration ranges of NaCl were 75 and 200 mM. The light-scattering autocorrelation functions of pure and MBP−Ag4-supplemented colloids and the sample treated with NaCl were also similar, but the time scale decay was found to be higher. The values of the hydrodynamic diameters based on PCS data were 17 nm for colloidal silver, 19 nm for the MBP−Ag4-treated sample, and 233 nm for the NaCl-treated sample. Therefore, the adsorption of an MBP−Ag4 protein shell to the surface of Ag NPs does not induce their aggregation and slightly alters their optical characteristics [172]. Likewise, Hamed et al. employed PCS analysis to characterize the silver and gold nanoparticles intended for pharmaceutical usage. The results indicated that the average diameter was 68 and 76 nm and the PDI value was 0.2 and 0.23 for Au and Ag NPs, respectively. The results indicated that the nanoparticles were synthesized with fairly well-defined size and greater homogeneity [173].

### 7.2. DLS

**Figure 4:**
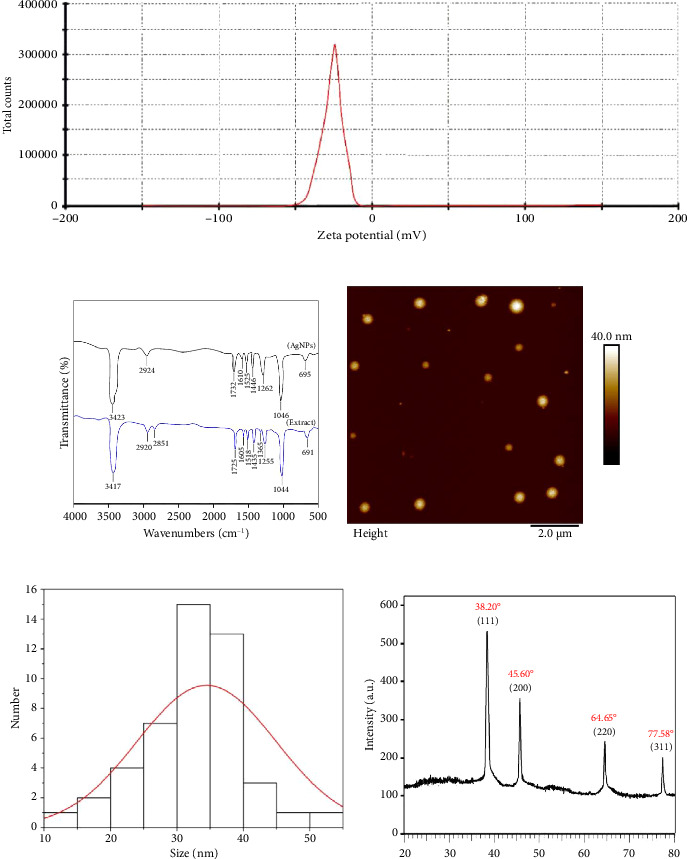
Physicochemical characterization techniques for silver nanoparticles (SNPs): (a) zeta potential of SNPs determined via dynamic light scattering (DLS) (reproduced with the permission of Elsevier), (b) FTIR analysis native casein and that bound to the nanoparticle surface has distinct differences in both shape and peak position (reproduced with the permission of Elsevier), (c) AFM micrograph demonstrating the size and morphology of SNPs (reproduced under Creative Common license), (d) size distribution of the designed SNPs, and (e) XRD pattern of the green-synthesized Ag NPs using the L. Roy Leana extract (reproduced with the permission of Elsevier).

DLS relies on the Brownian motion of scattered particles. In a liquid, particles move randomly in all directions, colliding continuously with solvent molecules, as described by Brownian motion principles. These collisions impart energy to the particles, resulting in their movement. Smaller particles are affected more significantly by energy transfer, which causes them to move faster than larger particles. By measuring the velocity of the particles and accounting for other factors that influence their movement, one can calculate the hydrodynamic diameter. The DLS technique estimates the average particle size and zeta potential of nanoparticles in suspension under various temperatures and pH conditions (Figure 4(a)). Zeta potential is used to evaluate the surface charge of the nanoparticles [174–176]. In a study conducted by Waghmare et al., we investigated the interaction between Ag NPs and BSA protein using DLS and other complementary techniques. These methods allowed them to quantitatively and qualitatively monitor the adsorption of BSA onto the Ag NPs. As the BSA attached to the Ag NPs, the average size of the particles, represented by the hydrodynamic radius, increased significantly from 24 to 35 nm. This growth eventually reached a plateau, indicating saturation. By analyzing this process of the BSA concentration, the researchers were able to distinguish between bound and unbound proteins. Notably, DLS measurements allowed them to determine the dissociation constant (*K*_*d*_) for the weakly bound protein layer (soft corona) to be 2.09 ± 0.30 μM [177]. Similarly, Ag NPs were designed by Lizoń et al. for immunoglobin-free light chain quantification. The metal nanoparticles were tested when covered with 10 particles of antibody and conjugated with 5–50 protein antigen particles. This formation of the second protein corona around the nanoparticles was validated by the increase in hydrodynamic diameter and zeta potential as per studies of DLS [156].

### 7.3. Atomic Force Microscopy (AFM)

AFM is a high-resolution type of scanning probe microscopy used to examine complex colloidal systems and characterize nanoscale objects, including nanoparticles ranging from 0.5 to over 50 nm, whether agglomerated or not. Discovered by Binning et al. in 1986, AFM is renowned for its ability to create and evaluate detailed images of surfaces at the atomic level. Since its introduction, AFM has simplified the analysis of both conducting and nonconducting materials, broadening its applications in nanotechnology. Researchers utilize AFM to gain insights across various fields, including electrochemistry, polymer science, biotechnology, material science, biophysics, and life sciences, particularly in understanding molecular and atomic-level changes and evaluating different statistical properties of nanoparticles using various analytical methods. In AFM, the force applied to a sample by a pointed probe tip is measured. The technique operates in three modes: contact, tapping, and noncontact. In contact mode, the probe tip maintains continuous contact with the sample surface while scanning, which can risk damaging both the tip and the surface. In contrast, tapping and noncontact modes do not physically touch the sample, making them more suitable for materials containing moisture or liquids [178, 179].

AFM has been utilized to greater effect by researchers worldwide for characterizing Ag NPs. In Figure 4(c), a representative AFM micrograph demonstrates the size and morphology of SNPs designed by Ortiz-Dosal et al. [180]. Liu utilized AFM to comprehend the behavior of Ag NPs when in contact with artificial cell membranes (supported lipid bilayers). The experiment determined the forces between the tip and the membrane by depositing Ag NPs on the tip of an AFM. The results showed a distinct trend of membrane repulsion. Surprisingly, the repulsion was significantly enhanced by the addition of HSA protein, possibly because the protein formed a layer on the surface of Ag NPs. Likewise, the protein coating also reduced the ability of the Ag NPs to pass through the membrane [181]. Drobota et al. studied the adsorption behavior of proteins on the surface of the Ag NPs. The research explained the protein's secondary structures, which in essence define the way the protein folds through AFM. These structures were compared in the presence and in the absence of Ag NPs which showed the influence of nanoparticles on the shape and behavior of the protein [182].

### 7.4. Scanning Electron Microscopy (SEM)

**Figure 5:**
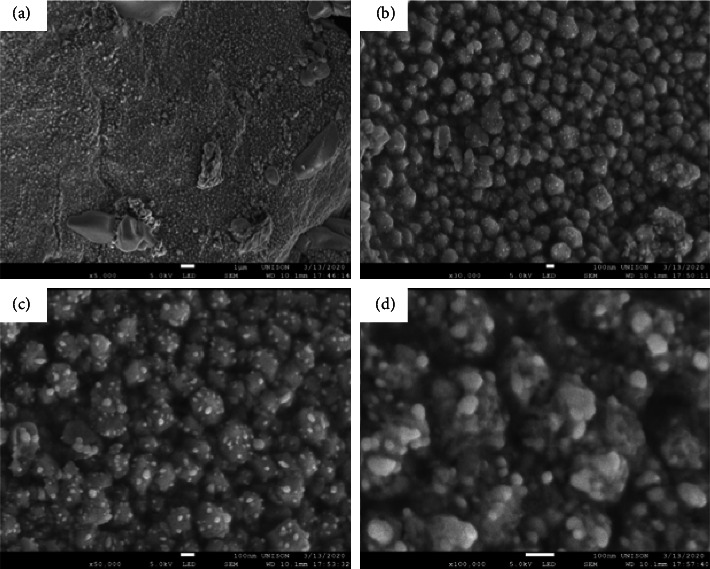
Representative SEM micrographs of SNPs analyzed at different magnification: (a) 5,000×, (b) 300,00×, (c) 500,00×, and (d) 1,000,00× (reproduced with the permission of Heliyon).

It helps to identify the signals that derive from electron–sample interactions that reveal information about the sample including external morphology (texture), chemical composition, crystalline structure, and orientation of materials making up the sample. It has a high-resolution type of fraction; more than 1000 times better than the optical diffraction and the particle surface was scanned under a high-energy beam [183]. A focused beam of electrons is utilized to image the specimen and gather information about its structure and composition. An electron source generates the electron beam, which is accelerated toward the sample using a positive electrical potential. Metal apertures and magnetic lenses are employed to constrain and focus the beam into a narrow, monochromatic stream. When the electrons interact with the atoms in the specimen, they produce signals that provide insight into the surface composition, topography, and other electrical characteristics of the specimen. These signals are analyzed to create images that reveal the topography (surface features), composition (elements and compounds present and their relative amounts), morphology (shape and size of the constituent particles), and crystallographic information (atomic arrangement). All of these properties of nanoparticles can be assessed using the SEM.  Figure 5 illustrates Ag NP analyses at different magnification levels by SEM for the morphology and size of the designed nanoparticles [65].

### 7.5. TEM

**Figure 6:**
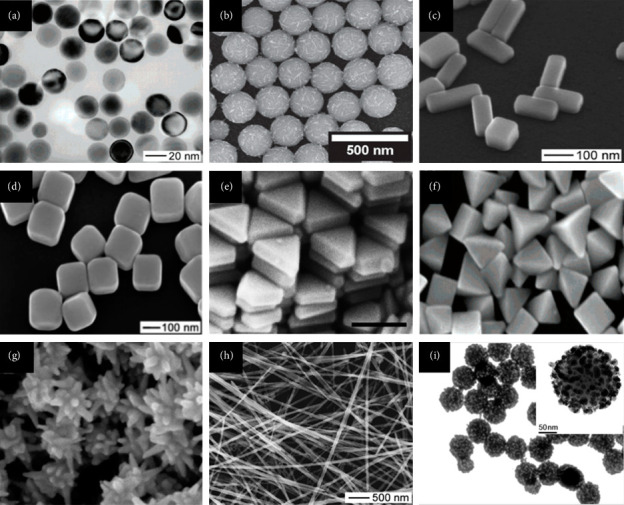
Electron microscopy images of the synthesized silver nanostructures reveal a remarkable diversity in size and morphology. This demonstrates the precise control achievable over reaction chemistry during synthesis: (a) silver nanosphere, (b) silver necklaces, (c) silver nanobars, (d) silver nanocubes, (e) silver nanoprism, (f) silver bipyramids, (g) silver nanostar, (h) silver nanocubes, and (i) silver nanoparticle embedded silica particle. All figures were reprinted with permission from the publisher of each article [184] (reproduced under an open-access Creative Common CC BY license).

TEM is a pivotal characterization technique for analyzing protein-based Ag NPs, offering high-resolution insights into their size, shape, and distribution. This technique employs a beam of electrons transmitted through a thin sample to create detailed images at the atomic or molecular level, essential for understanding the structural properties of nanoparticles as shown in Figure 6 [184]. TEM can reveal the crystalline structure and surface morphology of Ag NPs, enabling researchers to assess the effectiveness of protein capping agents in stabilizing and controlling nanoparticle growth. For instance, studies have shown that TEM images can depict the uniformity and dispersion of Ag NPs synthesized using various proteins, providing crucial data for tailoring their biomedical applications [185–187].

### 7.6. UV-Vis Spectroscopy

UV-vis spectroscopy is one of the essential characterization methods of protein-based Ag NPs. To assess the intensity of a UV-vis light beam before and after it interacts with a sample, the most straightforward approach is to position the sample between a light source and a detector. Measurements are conducted at each wavelength to identify the maximum absorbance, minimum transmittance, or lambda max value for specific wavelengths. The resulting data are plotted to show changes in absorbance and transmittance across different wavelengths. Each spectrum is corrected for background using a buffer blank to ensure that the sample's absorbance and transmittance do not include any spectral features from the buffer. The optical properties of metal plasmonic nanoparticles depend on factors such as size, shape, refractive index, interparticle distance, and their aggregation or disaggregation state. UV-vis spectroscopy plays a crucial role in characterizing these plasmonic nanomaterials [177, 188]. UV-vis spectroscopy can also be used to track the formation of Ag NPs synthesized with proteins as stabilizing agents since these have characteristic SPR absorption bands in the range of 400–450 nm [189]. It enables the observation of the progress of the synthesis process and ensures that nanoparticles are formed and the extent of aggregation or dispersion. Furthermore, the spectral data are useful in determining the relationship between the protein and Ag NPs, which will help in tweaking the synthesis parameters and guaranteeing replication. This approach is well described in scientific literature because of its simplicity, cost-effectiveness, and nondestructive nature which makes it standard in nanomaterial characterization [190].

### 7.7. Fourier Transform Infrared (FTIR) Spectroscopy

FTIR spectroscopy operates on the principle that when a molecule is exposed to IR light, it absorbs energy at frequencies specific to its structure. This absorption reveals detailed structural information about proteins in solutions with high spatial and temporal resolution [191]. FTIR spectroscopy is quite useful to investigate the secondary structural characteristics of proteins in different conditions. The amide functionality presents in most of the proteins possess characteristic absorption bands, such as the frequencies of the amide I (C=O stretch, 1600–1700 cm^−1^) and the amide II (C-N stretch and N-H deformation, 1530–1560 cm^−1^) bands, are associated with the protein structure. Likewise, the particular stretching and bending vibrations of the peptide backbone in the three regions of amide I, II, and III contain specifics about various types of secondary structures including alpha helix, beta sheets, turns, and random coils. Among all the amide bands of the peptide group, amide I is the most effective in indicating protein secondary structure. FTIR spectra (Figure 4(b)) of native casein and that bound to the nanoparticle surface have distinct differences in both shape and peak position, which indicates the alteration in the secondary structure of casein upon formation of the nanoparticles. The presence of a band at 1644 cm^−1^ in casein shows the unordered structures of the protein due to the presence of high proline content. In the case of casein–Ag NP conjugates, this band is shifted and it shows the content of the α-helix structure. The secondary amide peak at around 1514 cm^−1^ for native casein is also affected in the case of casein-stabilized Ag NPs. This can imply that the lone pair of electrons on the nitrogen of amide I and II regions may play a significant role in the formation/stabilization of Ag NPs. A band at around 1445 cm^−1^ corresponding to the δ CH of the CH_2_ group in native protein was also absent in casein [144].

### 7.8. X-Ray Diffraction (XRD)

This technique is used to provide information about the structure of a crystal at the atomic and molecular levels. The purpose of XRD is to investigate the structure of crystalline materials and also analyze their phase composition, crystallite size, shape, lattice, etc. [192]. For powdered samples, the XRD technique is the most suitable method. To quantify the composition of nanoparticles, researchers compare patterns from various diffraction database references with the positions and intensities of peaks obtained from XRD analysis. Identifying the phase of the sample material is the initial step in characterizing nanoparticles using XRD. The type of crystal can be determined by matching the intense XRD peaks with those in the existing library. Different XRD peak patterns reflect various atomic arrangements. When light of a specific wavelength strikes a periodic structure with a defined spacing, interference occurs, which is fundamental to the XRD technique. This principle is based on Bragg's law (*nλ* = 2*d*sinθ). Therefore, the analysis of the position, shape, and width of the diffraction peaks provides insights into shape anisotropy, crystalline phase, crystal size, and defects within the crystal [176]. A representative figure [174] for the results obtained via XRD of nanoparticles is illustrated in Figure 4(e).

## 8. Administration Routes of Protein-Based Nanoparticles for Drug Delivery

**Figure 7:**
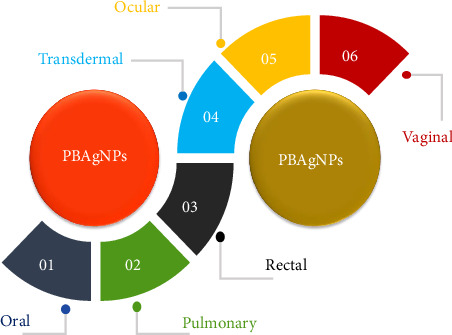
Illustration of various routes of administration for targeted delivery of protein-based silver nanoparticles.

Various proteins and peptides have been utilized for drug delivery in the modern segment of nanotechnology which is being used in many biopharmaceutical and therapeutic applications as shown in Figure 7. The choice of administration route has a substantial impact on the consequence of a drug. Some administration routes such as syringes and needles which are used for drug delivery are painful, costly, and require sterilization. As a result of which, researchers have adopted other noninvasive routes to deliver drugs via protein-based nanoparticles which are oral, transdermal, vaginal, pulmonary, and rectal [193, 194]. However, research is still focused on administration routes to establish successful delivery of drugs to target organs because of enzymatic barriers and chemical instability. To overcome this, there is a need for carrier proteins, diffusion parameters, and inhibitors to promote the bioavailability of drugs within the cell.  Table 2 demonstrates various noninvasive administration routes that researchers have adopted and focusing for future delivery use.

## 9. Applications of Protein-Based Ag NPs

Nanotechnology has sparked a great interest in all fields of science because of its high efficiency, biodegradability, and high compatibility in everyday life processes. Even today, diseases like cancer, HIV, tuberculosis, and several cardiovascular and respiratory pose a serious threat to mankind which propagates from generation to generation [206, 207]. By using nanotechnology and the modern use of nanoparticles, many serious diseases can be treated that are transferred genetically or have been occurring for decades [208]. The future of nanomedicine is bright, and researchers are focusing on implementing it in industrial-scale manufacturing because of its huge benefits in the field of biopharmaceuticals.

Focusing on nanomedicine, it helps in early diagnosis and prognosis, better treatment with good efficiency and high productivity is obtained. Some nanoparticles act as dyes which are helpful in bioimaging, and some as tags and label molecules that selectively target tumor cells [209]. Nanoparticles in molecular biology are gaining a lot of importance because of their tagging ability which can detect the genetic order of a sample. Furthermore, tissue engineering is a highly focused segment of nanotechnology in which tissues can be repaired, restored, and artificially created by using PBNs which can impact organ transplantation globally [210]. Nano-biosensors are also created which shed light on the origin of life. They have been utilized in the field of oncology and cancer diagnostics.

**Figure 8:**
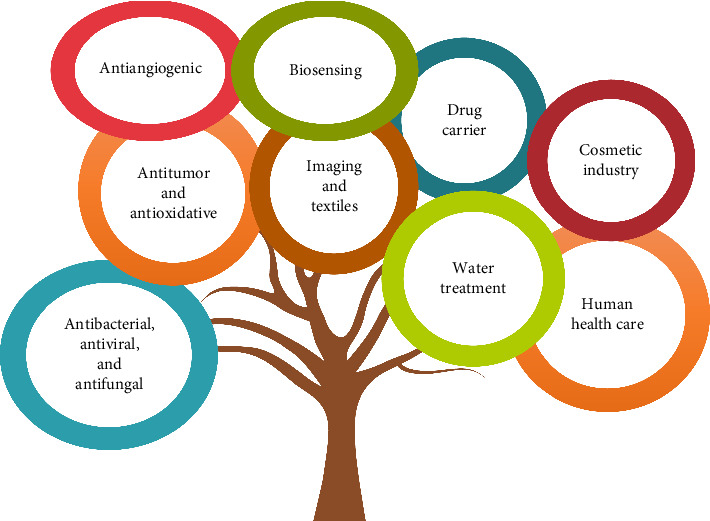
Graphical illustration of various applications of Ag NPs in biomedical, cosmetics, and bioimaging applied research.

Apart from health and medicine, nanotechnology is used in every aspect of life. From electronics to solar energy, and from traveling to space exploration, this branch of modern science has impacted us in every possible way. Nanotechnology helps in the storage of energy with conversion from one form to another and affects the production of renewable energy [206]. They are used for cheap energy production, catalysis of nanoparticles, and hydrogen equipment. Metallic nanoparticles play a great role in automobiles manufacturing by enhancing combustion of nanofilters and cleaning the exhaust manually by using metal nanoparticles as catalytic converters. Moreover, it promotes green technologies to purify our atmosphere from allergens and pollutants [211]. Nanorobots are created which play a vital role in the field of artificial intelligence. Using nanowires in highly conductive nanorobots, they can deliver drugs in vivo by selective targeting. Hydrophobic clothing has been developed which acts as water repellent because of the hydrophobic nature of nanoparticles used in it.  Figure 8 shows a graphical illustration of some applications of Ag NPs.

Researchers developed a new cancer therapy method, which uses a β-Elemene@Stanene nanodrug-delivering-drug system to engineer tumor-associated macrophages (TAMs) for cancer treatment. The strategy combines with chemoimmunotherapy to convert TAMs from M2 protumoral to M1 antitumoral cells which improves immune response against cancer cells. The therapeutic effects of nanodrug systems become stronger when silver-based nanomaterials are integrated into the systems. Ag NPs offer cancer therapy benefits through their antimicrobial and anticancer properties together with their capability to reshape immune system responses. Silver-based nanomaterials implement dual therapy when they deliver medicines with immunomodulators to cancer cells for simultaneous tumor cell recognition by the immune system [212].

### 9.1. Drug Delivery

Nanoparticles have impacted biomedicine to a great extent and brought a revolution in modern science. Biocompatible, biodegradable drugs are developed which are used for site-specific drug delivery. They have increased bioavailability, have lower side effects, avoid the body's defense mechanism, and avoid first-pass effect in most cases. They are cost-effective with reduced pain to patients. Drug delivery is achieved by carrier proteins, liposomes, or nanorobots for tumor targeting [64, 213–215]. Owoseni et al. reported the synthesis of Ag NPs through egg proteins. Such protein-coated Ag NPs can be used as drug carriers for hesperidin and other related drugs. The research revealed that the proteins got adsorbed on the Ag NPs and formed small, spherical, and negatively charged particles. Most significantly, when hesperidin was encapsulated onto these protein-Ag NPs, the antibacterial efficacy against the different bacterial species was enhanced. This study shows that the new approach of synthesizing protein-stabilized Ag NPs could be useful for delivering hesperidin and other drugs [216]. Similarly, Anandhakumar and Raichur developed a thin and translucent membrane for releasing drugs and proteins, once stimulated from outside the human body. The nature of the film's architecture enabled investigators to fill it with a drug (ciprofloxacin hydrochloride) and a protein (BSA). Ultrasound or laser light could then be used to open the film, to release the loaded cargo. The results showed a faster release of antibiotics in comparison with the protein because of its relatively smaller size. It is essential to note that despite simultaneous release, the structure and function of the protein remained intact throughout the procedure. Thus, drug delivery can occur in multiple ways; however, the development of this new film proved potential in eradicating colonization of *Staphylococcus aureus (S. aureus)*. Hence, the film could be applied on the skin like a patch for administering medication or vaccine, or a coated surface to prevent infections or inflammation on a medical device like an implant or a stent [217]. Moreover, recent research examined how serum SERS technology detects breast cancer through its implementation with thermally annealed Ag NP composite substrates. This study employed Ag NP properties for enhanced Raman scattering to analyze serum samples which aims to enhance both speed and accuracy of breast cancer screening [218].

### 9.2. Bioimaging

Nanotechnology has significantly contributed to the field of bioimaging. Nanoparticle images are used as contrast in MRI and ultrasound procedures. They are effective and highly useful in the diagnosis of different diseases such as cancer. Nanoparticles replace fluorescent dyes by promoting themselves as tag molecules. They have increased uptake as compared to fluorescent dyes because of their high permeability and low toxicity [219]. In a study conducted by Shagufta et al., a new, nontoxic green chemistry was deployed for the synthesis of Ag NPs (AgZE) by the reaction between silver nitrate (AgNO_3_) and the ethanolic leaf extract of *Zinnia elegans* (ZE). The cell viability assay of AgZE in normal cells (CHO, HEK-293T, EA.hy926, and H9c2) showed their biocompatible nature. Interestingly, the nanoparticles exhibited cytotoxicity toward different cancer cell lines (U-87, MCF-7, HeLa, PANC-1, and B16F10), while suppressing the formation of blood vessels which are necessary for tumor growth. Moreover, the tracking of AgZE nanoparticles in living mice was possible due to its inherent fluorescent nature under the near-IR light method (bioimaging). Hence, the study showed that AgZE can be a potential option for designing new cancer therapies and diagnostic agents [220]. Likewise, Maiti et al. investigated the surface-enhanced fluorescence phenomenon in biological dyes (fluorescein (FL)) by employing Ag NPs in an aqueous medium. In addition, density function theory (DFT) was deployed to study the electronic energy levels of different forms of FL. Notably, the dye conjugated with Ag NP/FL showed way relatively stronger fluorescence imaging of human lung fibroblast cells, as compared to FL dye alone. This highlights the significance of Ag NPs for high-resolution techniques such as human cell imaging and spectrophotometry [221].

### 9.3. Proteins and Peptide Delivery

Protein-based nanoparticles are useful for the treatment of various diseases. It is useful in delivering antigens and antibodies to treat various diseases. Its applications include the treatment of autoimmune disorders by delivering the required protein molecules via nanoparticles [222]. Nair et al. showed the bioconjugation of biologically important proteins such as cytochrome c and hemoglobin onto silver and gold nanosurfaces to study their surface binding chemistry. The research showed that the Ag NPs showed more aggregation tendency after hemin binding compared to the Au nanoparticles owing to the presence of carboxyl groups in hemin (Ag has more affinity toward –COOH groups), which makes particle interlinking feasible. On the other hand, the surface plasmon feature corresponding to Ag was broadened, indicative of aggregation due to greater interaction between particles [223].

Similarly, antimicrobial peptides (AMPs) have been potentially known for the treatment of microbial infections via topical or intravenous administration. However, due to several issues including their solubility (due to hydrophobic peptides), interactions with body components (e.g., with blood components when administered intravenously), susceptibility to environmental conditions other than proteases (e.g., degradation by light and oxidation), and a lack of targeting and controlled release of peptides, they cannot be administered in the solution form. Therefore, the development of a suitable drug delivery system for the effective delivery of AMPs is mandatory. Mei et al. designed and synthesized Ag NPs functionalized with AMPs to create an effective antimicrobial nanomaterial. In this study, bacitracin A (BA) and polymyxin E (PE), two potent AMPs targeting Gram-positive and Gram-negative bacteria, respectively, were integrated into the Ag NPs structure. The incorporation of AMPs significantly enhanced the antimicrobial activity of the Ag NPs. The minimum inhibitory concentration (MIC) values of the Ag NPs–BA and PE nanomaterial were determined to be 21.3, 10.3, 24, and 12.3 pmol/L against *E. coli*, *P. aeruginosa*, *S. aureus*, and *B. amyloliquefaciens*, respectively. These values were significantly lower than the MIC values of Ag NPs alone, which were found to be 207, 60, 133, and 72 pmol/L, respectively [224].

### 9.4. Cancer

Nanoparticles are highly applicable in oncology because of their nanosized scale. It helps in developing excellent bio images of cancer by using magnetic resonance imaging (MRI). Moreover, it accumulates easily in tumor cells with tumor-targeting drugs incorporated into it. Multiple techniques have been developed to cook tumor cells by radio waves that affect only nanoparticles and the neighboring cells adhering to it [225]. In such regard, Yaseen et al. synthesized bio-friendly gabapentin (GBP)-loaded Ag NPs with anticancer, antibacterial, and antifungal properties. The results showed a high level of efficacy of the synthesized GBP-Ag NPs against A549 lung cancer cells in comparison with controls, henceforth suggesting the potential use of GBP-Ag NPs as a nanodrug shortly [226]. In another study designed by Hanna et al., SNCs were synthesized as pH-sensitive, biodegradable carriers for oral intestinal delivery of 5-fluorouracil (anticancer drug). Surprisingly, the 5-FU encapsulation effectiveness inside all of the prepared SNC samples was substantially increased to 92.16 ± 0.57% with 3% Ag. Likewise, a slow and controlled release of the drug was observed at a pH of 7.4. The results were further validated by cytotoxicity assay and a high percentage of apoptotic cells (30.66%) within the treated HCT116 cell line, thus indicating the potential use of nanoencapsulation in oncology and cancer treatment [227].

### 9.5. Parkinson's Disease

Parkinson's disease is a condition recognized by the atrophy of the frontal cortex and ventricular expansion, as well as nerve cell loss in substantia nigra pars compacta (SNpc). On a molecular scale, the condition causes misfolding and accumulation of the α‐synuclein protein, hence compromising the synaptic plasticity and neurotransmitter release. The advent of nanotechnology has opened several avenues in different fields, particularly in nanomedicine. Due to the unique properties of NPs, they can be used as powerful contrast agents, which facilitate the delivery of drugs into the brain with the avoidance of the blood–brain barrier (BBB) via endocytosis, thus resulting in the successful treatment of neurodegenerative disorders [228].

In such regard, Dudhipala and Gorre synthesized Ropinirole (RP)-loaded RP-SLNs to investigate the oral and topical delivery of the drug. Furthermore, RP-SLNs were optimized and converted to hydrogel using Carbopol 934 as the gelling polymer, and pharmacokinetic and pharmacodynamics studies were performed on Wistar rats. The study showed sustained release and enhanced permeation of the RP-SLNs as compared to the controls in ex vivo and in vitro experiments. Overall, the results demonstrated that lipid nanoparticles and corresponding hydrogel formulations can be considered as an alternative delivery for the treatment of Parkinson's disease [229]. Likewise, Sardjono et al. synthesized silver-velvet bean (*Mucuna pruriens L*.) seed extract nanoparticles (Ag MPn) and evaluated its anti-Parkinson activity through the catalepsy test in mice. The research consisted of several stages, that is, extraction of velvet bean seed powder, synthesis and characterization of Ag MPn, and catalepsy test of Ag MPn. The results indicated that the intensity of catalepsy in mice treated with extract and the Ag MPn substantially decreased in comparison with the controls, thereby validating the role of nanotechnology in the combat of neurodegenerative disorders [230].

### 9.6. Alzheimer's Disease (AD)

The condition is associated with hippocampus degeneration, which in turn leads to the alteration of synaptic connections due to the formation of plaques constituted by amyloid proteins. At the molecular level, the brain tissue in AD is characterized by the combined presence of two classes of abnormal, insoluble, and highly dense structures: extracellular amyloid plaques and intraneuronal neurofibrillary tangles. The soluble component of these structures is the Aβ peptides for plaques and tau (*τ*) proteins for tangles [231]. Early diagnosis and treatment of AD are achieved by techniques that are highly specific for brain endothelial cells. Considering the permeability of nanoparticles to cross through the BBB and deliver drugs, these nanocomposites can be considered as a potential option for eliminating amyloid burden. Nano-biosensors are also made to detect early biomarkers identifying the subject which may evolve into AD [232]. To further investigate the role of Ag NPs in the treatment of Alzheimer's, Zhang et al. utilized an aqueous extract of *N. khasian*a leaf as a reducing and stabilizing agent for the synthesis of Ag NPs and assessed their therapeutic potential in sporadic AD rats produced by intracerebroventricular injection of streptozotocin (i.c.v.–STZ). The results showed Ag NPs prevented the effect of deficits in recognition and spatial memory of STZ-induced rats. This implies that these specific Ag NPs have the potential to be used in the management of AD [233]. Similarly, Parveen et al. synthesized Ag NPs from the extract of *Acacia auriculiformis (AA*) leaves using a biogenic approach. Considering the smaller size of Ag NPs validated via TEM/SAED and SEM-EDX, these nanostructures were further taken for biological studies. The study established that these nanoparticles were thermally stable and were found to prevent the aggregation of proteins responsible for Alzheimer's and Parkinson's diseases. This holds great promise for these Ag NPs in the development of new theranostics for protein-associated diseases [234].

### 9.7. Tuberculosis

The disease is caused by an airborne pathogen, *Mycobacterium tuberculosis* (MTB) that primarily affects the lungs and leads to severe cough, chest pain, and fever. Novel antibiotics are introduced into the market to overcome the prolonged medication treatment for tuberculosis. Avoidance of steroids and medications that cause side effects is eluded. More compliant and effective drugs are developed which are of great importance in delivering antituberculosis drugs to the specific site leading to a more suitable and effective way to treat this infectious disease [235, 236]. In such regard, Kalmantaeva et al. designed Ag NPs against the MTB H37Rv strain and subsequently analyzed their antibacterial properties. The study was performed in a murine model affected by chronic tuberculosis. The results showed that the administration of Ag NPs via inhalation at a dose of 0.1 mg/kg to tuberculosis-infected mice resulted in a twofold decrease in the colonization of the lungs and spleens by *M. tuberculosis*. In these animals, the quantity of protein in the bronchopulmonary lavage fluid was reduced by two times, which indicates a decrease in the inflammatory processes in the lungs. Likewise, after the introduction of Ag NPs, a recovery in the ratio of lymphocyte populations in the spleen and cytokine balance was also observed [237].

On the contrary, Sarkar et al. showed the potential health risks associated with the Ag NPs present in various consumer products. The study revealed that the uptake of Ag NPs by macrophages may lead to alterations in innate immune functions. Furthermore, exposure of monocyte-derived macrophages (MDM) to Ag NPs significantly reduced cellular viability and macrophage survival, thus negative influence on the immune cells to combat *tuberculosis*. The results also showed an overall increase in the stress levels of macrophages, hence limiting their ability to fight the infection. The study highlighted the controversial role of Ag NPs as modulators of infectious pathogen-induced immune response [238].

Researchers introduced NIR-II AIE Luminogen-Based Erythrocyte-Like Nanoparticles with Granuloma-Targeting and Self-Oxygenation Characteristics for Combined Phototherapy of Tuberculosis in their research article. The research presents biomimetic nanoparticles that behave like erythrocytes to extend blood retention and direct delivery to TB granulomas. The nanoparticles hold aggregation-induced emission (AIE) luminogens and Prussian blue nanoparticles which enable both photodynamic therapy (PDT) and photothermal therapy (PTT) treatments. The AIE photosensitizers create reactive oxygen species upon laser stimulation which destroys MTB bacteria simultaneously with the Prussian blue nanoparticles that produce hyperthermic conditions for bacterial elimination. Nanoparticles possess a self-oxygenation mechanism that helps improve PDT effectiveness by remedying the hypoxic conditions present in granulomas. Studies using mice confirmed that combined therapeutic methods led to granuloma inhibition and diminished bacterial survival which could represent a new way to treat TB apart from antibiotics [239].

### 9.8. Dentistry

Creation and utilization of nanoscale materials act at the atomic, molecular, and supramolecular levels within the dentistry segment. Nanofilled resin components are developed that offer wear resistance, strength, and aesthetics due to their luster retention and enhanced polish ability. Nanofillers are developed which resemble hard-connective tissues such as dentin and enamel [240]. In a prospective controlled clinical trial conducted by Dos Santos et al., a new anticaries agent, nanosilver fluoride (NSF) was employed to cure caries among children. The composition of NSF constituted Ag NPs fluoride and chitosan. The results showed that the treatment with NSF was comparatively more effective in arresting the progression of caries and maintaining its levels in two-thirds of the cases [70]. Similarly, Salas-López et al. incorporated Ag NPs into dental sealants to evaluate the effects on dental caries. The results showed silver nanoparticle-mixed sealant reduced tooth demineralization significantly and likely increased remineralization, compared to the conventional sealant. Based on such evidences, it is possible to state that Ag NPs may be used as an effective alternative against tooth decay [241].

### 9.9. Ophthalmology

The use of nanoparticles has been instrumental in treating oxidative stress, reducing intraocular pressure, and preventing scarring in choroidal vessels following glaucoma surgery. To date, among various applications of nanomedicine, the development of eye ointments using nanoemulsions, for the treatment of dry eyes, holds a prime significance in restoring blurred vision, cornea, and conjunctivital disorders [242, 243]. In such regard, Luo et al. devised a new treatment using silver particles capped with gelatin (G-Ag NPs) against *S. aureus* (a pathogen that causes keratitis). The results showed that these G-Ag NPs were more stable and efficient at reducing bacterial growth than the uncoated particles. In rabbit studies, the G-Ag NPs applied directly to the eye helped clear the infection and prevented abnormal growth of the blood vessel, therefore suggesting that G-Ag NPs could be a promising new treatment for eye infections caused by *S. aureus* [244]. Likewise, Nguyen et al. proposed a biomedical strategy to formulate Ag NPs as intrinsically therapeutic agents for the treatment of *S. aureus.* The study showed substantial influence of particle size of Ag NPs, as increased size particles (37.2 ± 5.3 nm) exhibited better ocular biocompatibility and stronger antiangiogenic activity, but poorer bactericidal performance, whereas smaller particles (3.3 ± 0.7 nm) showed prominent bactericidal activity but at the expense of risking eye damage. Hence, medium-sized Ag NPs (15.0 ± 3.6 nm) were proven as the best-suited option for significantly improving the corneal recovery. This study highlights the importance of designing nanoparticles with specific properties for effective and safe treatments [245].

### 9.10. Tissue Engineering

Applied nanotechnology is used to repair and regenerate tissues that are damaged or scarred by using various protein-based nanoparticle systems in the form of skin ointment including collagen and keratin, and the injuries were healed with controlled drug delivery. Artificial organ implants and artificial cell proliferation can be achieved using nanotechnology which can lead to life extension [246]. To investigate the role of SNCs in bone tissue engineering, Marisch et al. prepared an alginate/HA composite scaffold by internal gelation. This was followed by a freeze-drying procedure to obtain a porous structure. Subsequently, nanoparticles were prepared in the presence of a lactose-modified chitosan and this colloidal solution was adsorbed on the scaffolds by exploiting electrostatic interactions. The results showed that silver had no deleterious effects on the osteoblast proliferation, while at the same time, these nanocomposites exerted strong bactericidal pressure on both Gram-negative and Gram-positive strains. This suggests that these new scaffolds could be a valuable tool for bone grafting procedures [247]. Similarly, in another study, Srivastava et al. prepared tasar fibroin nanofibrous mats using 1-butyl-3-methylimidazolium acetate for skin tissue engineering. The mats were then coated with Ag NPs in situ using dandelion (*Tridax procumbens*) leaf extract. The results showed significant metabolic activity of L929 fibroblast cells seeded Ag NPs-coated tasar nanofibrous mat (Ag NP-TNF-25) indicating the growth and viability of the cells [248].

The therapeutic benefits of protein-based Ag NPs have been established for bone metastasis treatment because of their specialized characteristics. Scientific engineering of these nanoparticles allows them to deliver therapeutic proteins straight to bone tissues with reduced systemic side effects while achieving better treatment performance. Research has proven that Ag NPs act as regulators of mesenchymal stem cell functions for bone regeneration while showing detrimental effects against bone cancer cells which make them suitable for bone tissue engineering and cancer treatment applications [249]. Research shows that collagen-based Ag NPs demonstrate suitable compatibility with human tissue and skin penetration abilities which lead to possible uses in drug delivery systems targeting bone conditions [250]. Bioengineered Ag NPs with BSA caps show anticancer and apoptotic effects against different cancer cells such as bone cancer cells, thereby indicating their potential to treat bone metastases [132].

### 9.11. Respiratory Diseases

Different allergic, genetic, and infectious diseases can be treated using nanoparticles in focus. It reduces lung inflammation and airway hyperactivity by using antigen-based nanoparticles. In addition to this, it also reduces allergen-induced airway inflammation by promoting restoration of lung morphology by reduction in inflamed cells [251]. This was further validated by Holmila et al. by studying the role of Ag NPs in the treatment of lung cancer. The results showed that the exposure to Ag NPs caused cell cycle arrest and decreased cell proliferation in lung cancer cell lines including A549, BEAS-2B, and Calu-1, but not in NCI-H358 followed by an increase in oxidative stress levels. Hence, this suggests that Ag NPs may form complex interactions with cells and therefore need further investigation before their effective usage in the treatment of lung cancer [252]. In another study, Seiffert et al. explored the role of silver particles (Ag NPs) of varying sizes and coating to study their effect on murine lungs. The results showed that these nanoparticles can potentially trigger inflammation depending on the type of rat and the Ag NP properties. Smaller particles were found to cause a stronger inflammatory response than larger particles, while the specific coating on the particles showed insignificant effects. In some rats, the inflammation resembled asthma, with both increased mucus and airway tightening. Thus, the study highlighted underlying issues related to the inhalation of Ag NPs, which can become a potential health hazard [253].

## 10. Drawbacks and Challenges

Proteins are natural biomolecules, which differ in their composition and character resulting in a diverse range of size distribution of nanoparticles which cause variations during analysis. As a result, some problems may occur in preparing it on an industrial scale. To tackle this problem, researchers have adopted recombinant protein technology [254]. The benefits of using this technology include precise preparation, predictability of cross-linking moieties, and monodispersed which makes them an ideal candidate for tissue engineering and drug delivery.

Studying the safety of Ag NPs is trickier than testing traditional chemicals. Standard tests for cell health can be misleading because Ag NPs tend to interfere with the applied light measurements [255]. Additionally, factors such as size, shape, and surface coating of Ag NPs can also drastically affect their toxicity profile [256]. Therefore, it is essential to analyze Ag NPs throughout an experiment, as they may agglutinate making them less likely to be absorbed by cells [257]. Despite these challenges, the unique optical properties of Ag NPs can be helpful for research. Researchers often use light to track Ag NPs inside cells and visualize their site of accumulation in the body [258]. This can be useful to understand how Ag NPs work and how long they last. In short, although difficult, studying the safety of Ag NPs is important. Their special properties may be challengeable on one hand, but they also offer unique opportunities for researchers to comprehend how such particles interact with living things [259].

The present research is also focused on animal proteins, to overcome their hydrophilic nature which solubilizes rapidly in aqueous solvents. Their hydrophilic properties cause them to swell and burst, releasing the drug into the environment. The use of cross-linkers to modify the structure of proteins such as glutaraldehyde and formaldehyde may remain unreacted within the surface of nanoparticles and can cause toxicity during degradation in vivo. In such regard, plant proteins are preferred over animal proteins because they do not need any cross-linkers for structure modification [260].

## 11. Future Perspectives

Protein-based Ag NPs are emerging as an innovative and sustainable alternative in the field of nanotechnology, due to their exceptional properties and their ability to provide more environmentally friendly and biocompatible solutions compared to conventional Ag NPs. These nanoparticles are synthesized through a “green” process, using proteins as reducing and stabilizing agents, which makes them safer and more environmentally friendly. This technique offers several advantages, such as the ability to control the size and shape of the nanoparticles, which is crucial for enhancing their effectiveness across a wide range of applications. The biocompatibility of proteins also makes protein-based Ag NPs more suitable for medical uses, reducing the risk of toxicity in living organisms. Research on metal-organic frameworks (MOFs) has risen for biomedical applications because their adjustable porosity and large surface area make them ideal for effective drug loading and delivery. Ag NPs maintain superior advantages compared to MOFs when considering specific application areas. The antimicrobial efficacy of Ag NPs remains strong because they efficiently eliminate pathogens including resistant strains. The antimicrobial effect of silver ions occurs when these ions breach microbial cell membranes and cause the destruction of vital cellular functions which results in rapid microbial death [261].

The synthesis process for Ag NPs remains simple because scientists can use multiple methods including green synthesis which converts silver ions into nanoparticles with biological organisms. Ag NP synthesis methods provide flexibility for controlling both sizes and shapes of nanoparticles which produce materials suited for particular uses [262].

Ag NPs demonstrate plasmonic properties which allow them to function in biosensing applications and imaging systems. Ag NPs function exceptionally well as surface-enhanced Raman scattering (SERS) detection probes because they amplify electromagnetic fields to achieve precise molecular diagnostic analysis [263].

The catalytic properties of Ag NPs enable them to perform efficiently in reduction reactions, as well as oxidation reactions and carbon–carbon bond-forming processes. The combination of high surface-to-volume ratio and active surface atoms makes Ag NPs highly efficient catalysts for therapeutic applications that need catalytic processes [263]. The combination of MOFs and Ag NPs brings tunable porosity along with high drug-loading capabilities, but Ag NPs provide better antimicrobial properties, simpler synthesis methods, and distinctive optical properties and catalytic capabilities for specific biomedical applications [263].

In the medical field, protein-based Ag NPs hold significant potential for antimicrobial applications, which could revolutionize the treatment of bacterial and fungal infections. These nanoparticles can be used as more effective antibacterial agents, helping to reduce antibiotic resistance. Additionally, when stabilized by proteins, their biocompatibility increases, making them suitable for controlled drug delivery and targeted medication administration, improving treatment efficacy while minimizing side effects. They are also being explored in diagnostics, serving as components of highly sensitive biosensors for detecting disease biomarkers, such as cancer, and in biomedical imaging, acting as contrast agents to enhance MRI or positron emission tomography techniques.

In agriculture, protein-based Ag NPs could offer innovative solutions for crop protection, acting as natural antimicrobial agents that combat pathogenic bacteria and fungi without the adverse effects of traditional pesticides. Furthermore, their ability to enhance nutrient absorption in plants could contribute to higher productivity and sustainability in agriculture, reducing the reliance on chemical fertilizers. In the industrial sector, protein-based Ag NPs are being researched for use in gas sensors and catalysts, opening up applications in environmental monitoring, energy conversion, and pollutant removal.

However, despite their advantages, significant challenges remain in the widespread adoption of protein-based Ag NPs. One of the main obstacles is the scalability of their production. While the synthesis of these nanoparticles is promising at a small scale, large-scale production remains a challenge due to the complexity of the processes involved and the cost associated with producing high-quality proteins. Additionally, the stability of the nanoparticles in biological environments is a concern, as they may undergo aggregation or degradation, which could limit their effectiveness in practical applications. There also remains a need for a thorough evaluation of their toxicity and safety over the long term, particularly in medical and agricultural applications, to ensure they do not pose a risk to human health or the environment. Lastly, the costs associated with protein production and the need to optimize manufacturing processes are factors that must be addressed to make large-scale commercialization feasible.

## 12. Conclusion

Nanotechnology is transforming everyday life, driving advancements across health, medicine, transportation, energy, environmental sustainability, and even space exploration. This review highlights the synthesis of protein-based Ag NPs and their pivotal role in nano-biosciences and nanotechnology. The integration of nanomedicine, particularly through protein-based nanoparticles for drug delivery and tissue engineering, represents a revolutionary approach with far-reaching implications. Silver, when conjugated with proteins, enhances tumor-targeting efficiency due to its antiangiogenic, antibacterial, and antifungal properties. Beyond oncology, these nanoparticles show promise in bioimaging and food nanotechnology, further expanding their impact across scientific and industrial domains.

Protein-based nanoparticles offer a wide range of therapeutic agents that exhibit excellent characteristics as a carrier protein for drug delivery. It plays a major role in the diagnosis, treatment, and prognosis of various diseases, with little or no side effects. Nanotechnology has progressed immensely in the field of oncology by incorporating different drugs into protein-based nanoparticles for tumor targeting. Different routes of administration have been adopted in the form of inhalers and ointments to avoid the first-pass effect and to maximize the delivery of drugs to the target organ. However, much research is needed to understand the biologics of drug delivery systems, their interaction with the environment, their phenomenon of absorption, and their release from the body without causing any toxicity. The role of nanotechnology in the field of gene therapy is an ideal approach to treat genetic diseases. The upcoming future contains great potential for nanomedicine, but extensive research is needed to apply it in practical life.

## Figures and Tables

**Table 1:** Summary of different protein-based nanoparticles and their biological source, properties, and their functions.

Proteins	Biological source	Mean size of nanoparticles	Advantages	Properties and functions	References
Gelatin	Hydrolysis of fibrous, insoluble protein collagen which is obtained from the skin, bones, and connective tissues	50–200 nm	Nontoxic, noncarcinogenic, low antigenicity, ideal for parenteral formulations. Easily cross-linked, readily sterilized, cheap in nature, no contamination with pyrogens	Gelatin Type A has pH 7–9 while gelatin Type B has pH 4–5. Prepared as microspheres and nanoparticles	[76, 77]
Albumin	Bovine serum albumin (BSA) is obtained from egg white. Human serum albumin (HSA) obtained from human blood plasma	10–200 nm	Highly biocompatible, biodegradable, and nonimmunogenic, preferred for targeted drug delivery	Nontoxic water-soluble protein, which is easy to prepare, and easily attachable via covalent linkage. Prepared as nanospheres and nanocapsules	[78, 79]
Collagen	Abundantly present in vertebrates. Highly enriched in bones, tendons, and skin	64–84 nn	Biodegradable, biocompatible, weak antigenicity, and insoluble in organic solvents. Helps in the formation of blood vessels, skin texture, bones, and cartilage	Water-soluble protein which is a suitable carrier for drug delivery in the form of nanoparticles	[80, 81]
Gliadin and legumin	Gliadin is a gluten protein extracted from wheat. Legumin (vegetable casein) is obtained from beans, lentils, and peas	5–120 nm	Biocompatible, biodegradable, naturally occurring compound which is nontoxic and stable, of hydrophobic nature	Gliadin is a suitable polymer for the oral and topical drug delivery system. Used for the preparation of mucoadhesive formulation because it can adhere to the mucus membrane	[82–84]
Elastin	Abundantly found in connective tissues, extracellular matrix	20–743 nm	Maintain elasticity and tensile strength of the tissues	Elastin nanoparticles are an ideal source for drug delivery which are prepared via electrospraying technique. Several drugs can be loaded which shows sustained drug release in vitro	[85, 86]
Zein	It consists of rich prolamin protein that contains hydrophobic amino acids, proline, and glutamine. Obtained from corn and corn gluten meal	170–275 nm	Safe, compatible, cheap, adhesive, and possess great value as a biomaterial. Also, it is nontoxic, stable, and biodegradable	Nanoparticles formed from zein proteins have been prepared to encapsulate several drugs and bioactive compounds including coumarin and 5-fluorouracil. Also highly used for films and coating	[87–89]
Soybean	Obtained from plant sources in enriched form of soy protein	15–80 nm	Highly acceptable in nutraceutical and pharmaceutical areas, effective in drug encapsulation because of its hydrophobic nature	The important component for soy protein isolate is glycinin and *β*-conglycinin. Upon addition of cross-linking agent's soy protein isolate form aggregate and at certain temperature microspheres, hydrogels and polymer blends were formed conglycinin	[90, 91]
Milk proteins	β-Lactoglobulin (BLG) and casein. Obtained from milk sources	100–200 nm	Biocompatible, biodegradable, nontoxic, easily digested, prevents muscular breakdown, ideal candidate for drug delivery	The beta-lactoglobulin consists of two disulfide bonds and one free thiol group. BLG has good gelling ability which was used as drug delivery application. Casein exists as micelles size of ranges of 100–200 nm, and it used to transport the calcium and amino acid. Micelles of calcium have no fixed structure due to changes like temperature, pH, ionic strength, and water activity occur. Moreover, it can withstand heat and mechanical forces	[92, 93]
Silk proteins	Natural polymer obtained from silkworms and spiders	35–1200 nm	Highly biodegradable and biocompatible, with improvement of cell adhesion and proliferation and excellent cross-linking makes it an ideal carrier for drug delivery as a nanomaterial	Protein-based macromolecule which normally contains fibroin and sericin is used extensively in the form of hydrogels, scaffolds, electro-spun fibers and spheres	[94]
Lectins	Obtained in rich amounts from sea foods and legumes	50–200 nm	Low immunogenicity, high bio adhesion, high binding affinity	They are sugar-binding proteins that form glycoconjugates having high bioadhesion and effective drug delivery to the target site	[95, 96]

**Table 2:** Representation of different administration routes of protein-based nanoparticles.

Routes of administration for protein-based nanoparticles	Advantages	Applications	References
Oral	The preferred, noninvasive route offers patient convenience, compliance, and efficient drug delivery	Cheaper to produce and does not need sterilization during manufacturing. Have similar parenteral formulations as compared to normal drugs such as the delivery of insulin via protein-based nanoparticles	[195, 196]
Pulmonary	A noninvasive route is used to deliver the drug to the target organ. Possess huge potential for sustained drug delivery via nebulizers and inhalers. Avoids first-pass effect	Inhalation of colloidal systems has been extensively studied with a lot of promise. Offers many advantages such as high vascularization and sustained release. Various lung infections have been treated by incorporating protein nanoparticles with antibiotics in vitro studies	[197, 198]
Rectal	Highly vascularized, avoids presystemic metabolism, ideal for drugs that cause nausea/vomiting or cause inflammation in the GI tract, a large dose can be administered with targeting the lymphatic system	The ideal dosage for systemic drug delivery includes gels which offer optimum balance. Factors that affect this route of administration are the amount of fluid present in the rectum with high pH and the buffer capacity of the rectal fluid	[199, 200]
Transdermal	Offers many benefits such as better patient compliance and evasion of the first-pass effect. However, it has been difficult for researchers to discover drugs that are small and lipophilic enough to permeate through the skin	Nanosized titanium oxide and zinc oxide are used as sunscreens and sun protectants to avoid harmful sunlight radiation. Immunosuppressive calcineurin and corticosteroids are used to treat different types of dermatitis	[201, 202]
Ocular	Nanotechnology has offered new perspectives in managing ocular diseases by controlling the release of drugs, low eye irritation, improving drug availability, and enhancing ocular tissue compatibility	Ocular systems have been developed in the form of eye drops to treat certain kinds of diseases such as corneal injury, dry eye, conjunctivitis, keratitis, and cataracts. Several approaches have been employed for ocular clinical diagnosis such as optical coherence tomography, fundus photography, fluorescein angiography, and positron emission tomography	[203, 204]
Vaginal	This route offers many advantages such as bio adhesion, easy penetration of mucosa, and controlled release which decreases side effects caused by pharmaceutical drugs	This route of administration offers the treatment of factors associated with female reproductive tract, cancer, sexually transmitted diseases, and fungal and bacterial infections	[205]

## Data Availability

The data that support the findings of this study are available from the corresponding author upon reasonable request.
